# DE-PASS Best Evidence Statement (BESt): A Systematic Review and Meta-analysis on the Effectiveness of Trials on Device-Measured Physical Activity and Sedentary Behaviour and Their Determinants in Children Aged 5–12 Years

**DOI:** 10.1007/s40279-024-02136-8

**Published:** 2024-12-06

**Authors:** Mohammed Khudair, Anna Marcuzzi, Gavin Daniel Tempest, Kwok Ng, Ratko Peric, František Bartoš, Maximilian Maier, Mirko Brandes, Angela Carlin, Simone Ciaccioni, Cristina Cortis, Chiara Corvino, Andrea di Credico, Patrik Drid, Francesca Gallè, Pascal Izzicupo, Henriette Jahre, Athanasios Kolovelonis, Atle Kongsvold, Evangelia Kouidi, Paul Jarle Mork, Federico Palumbo, Penny Louise Sheena Rumbold, Petru Sandu, Mette Stavnsbo, Ioannis Syrmpas, Sofia Vilela, Catherine Woods, Kathrin Wunsch, Laura Capranica, Ciaran MacDonncha, Fiona Chun Man Ling, DE-PASS

**Affiliations:** 1 School of Psychology, Cardiff University, Cardiff, UK; 2 Department of Public Health and Nursing, Norwegian University of Science and Technology (NTNU), Trondheim, Norway; 3 Department of Sport, Exercise and Rehabilitation, Northumbria University, Newcastle Upon Tyne, UK; 4 Faculty of Education, University of Turku, Turku, Finland; 5 Department of Physical Education and Sport Sciences, Physical Activity for Health Centre, Health Research Institute, University of Limerick, Limerick, Ireland; 6 Institute of Innovation and Sports Science, Lithuanian Sports University, Kaunas, Lithuania; 7 Exercise Physiology Laboratory, OrthoSport Banja Luka, Banja Luka, Bosnia and Herzegovina; 8 Department of Psychology, University of Amsterdam, Amsterdam, Netherlands; 9 University College London, London, UK; 10 Department of Prevention and Evaluation, Leibniz Institute for Prevention Research and Epidemiology (BIPS), Bremen, Germany; 11 Centre for Exercise Medicine, Physical Activity and Health, Sport and Exercise Sciences Research Institute, University of Ulster, Jordanstown Campus, Coleraine, UK; 12 Department of Wellbeing, Nutrition and Sport, Faculty of Human Sciences, Education and Sport, Pegaso Telematic University, 80143 Naples, Italy; 13 Department of Movement, Human and Health Sciences, University of Rome “Foro Italico”, 00135 Rome, Italy; 14 Department of Human Sciences, Society and Health, University of Cassino and Lazio Meridionale, Cassino, Italy; 15 Department of Psychology, Università Cattolica del Sacro Cuore, Milan, Italy; 16 Department of Medicine and Aging Sciences, University “G. d’Annunzio” of Chieti-Pescara, Chieti, Italy; 17 Faculty of Sport and Physical Education, University of Novi Sad, Novi Sad, Serbia; 18 Department of Medical, Movement and Wellbeing Sciences, University of Naples Parthenope, Naples, Italy; 19 Department of Physiotherapy, Oslo Metropolitan University, Oslo, Norway; 20 Department of Physical Education and Sport Science, University of Thessaly, Volos, Greece; 21 Laboratory of Sports Medicine, Department of Physical Education and Sports Science, Aristotle University of Thessaloniki, Thessaloniki, Greece; 22 National Institute of Public Health in Romania, Bucharest, Romania; 23 Department of Sport Science and Physical Education, Faculty of Health and Sport Sciences, University of Agder, Kristiansand, Norway; 24 Laboratory for Integrative and Translational Research in Population Health (ITR); EPIUnit, Institute of Public Health, University of Porto, Porto, Portugal; 25 Institute of Sports and Sports Science, Karlsruhe Institute of Technology, Karlsruhe, Germany

## Abstract

**Background:**

To combat the high prevalence of physical inactivity among children, there is an urgent need to develop and implement real-world interventions and policies that promote physical activity (PA) and reduce sedentary behaviour (SB). To inform policy makers, the current body of evidence for children’s PA/SB interventions needs to be translated.

**Objectives:**

The current systematic review and meta-analysis aimed to identify modifiable determinants of device-measured PA and SB targeted in available intervention studies with randomized controlled trial (RCT) and controlled trial (CT) designs in children and early adolescents (5–12 years) and to quantify the effects of the interventions within their respective settings on the determinants of PA/SB and the outcomes PA and SB.

**Methods:**

A systematic search was conducted in MEDLINE, PsycINFO, Web of Science, SPORTDiscus and CENTRAL. Studies were considered if they were randomized controlled trials (RCTs) or controlled trials (CTs), included children and/or early adolescents (5–12 years; henceforth termed children), measured PA and/or SB using device-based methods and measured PA and/or SB and determinants of PA/SB at least at two timepoints. Risk of bias was assessed using the Cochrane Risk of Bias Tool for Randomised Trials (RoB2) for RCTs and Risk of Bias in Non-randomised Studies of Interventions (ROBINS-I) for CTs. The quality of the generated evidence was assessed using Grading of Recommendations Assessment, Development and Evaluation (GRADE). Robust Bayesian meta-analysis was conducted to quantify the effects of the interventions on the determinants of PA/SB, and the outcomes PA and SB, stratifying by study design, duration of PA/SB measurement, intervention setting and duration of follow-up measurement. Study characteristics and interventions were summarized.

**Results:**

Thirty-eight studies were included with a total sample size of *n* = 14,258 (67% girls). Settings identified were school, family/home, community and combinations of these. The review identified 38 modifiable determinants, spanning seven categories on individual, interpersonal and physical environmental levels, with 66% of determinants on the individual level. Overall, the results indicated trivial-to-moderate effects of the interventions on the determinants of PA and SB, with mostly trivial level of evidence for the presence of an effect (as indicated by a small Bayes factor; BF_10_ < 3.00). The exceptions were moderate effects on parental PA modelling in the family/home setting and SB measured during specific parts of the school day. Higher quality of evidence was found in the family/home setting compared with other settings.

**Discussion:**

Overall, the results indicated that interventions have neither been effective in modifying the determinants of PA/SB, nor changing the PA/SB outcomes in children. In general, the approach in the current review revealed the breadth of methodological variability in children’s PA interventions. Research is needed to address novel approaches to children’s PA research and to identify potential determinants to inform policy and future interventions.

**Registration:**

International prospective register of systematic reviews (PROSPERO): CRD42021282874.

**Supplementary Information:**

The online version contains supplementary material available at 10.1007/s40279-024-02136-8.

## Key Points

**Table:** 

The results of the current systematic review and meta-analysis indicated that interventions have not been effective in promoting physical activity and reducing sedentary behaviour. The effects on the determinants of physical activity/sedentary behaviour were mixed but overall small.
The family/home setting showed overall higher quality of evidence as assessed by GRADE. Determinants involving parents, including co-physical activity, parental physical activity modelling and parenting for physical activity, showed promise.
The current systematic review and meta-analysis highlighted the methodological variability in physical activity interventions and the continued need for theory-based interventions. Gaps were identified relating to policy-based factors on the environmental level and individual level.

## Introduction

Physical inactivity contributes to the rising global obesity crisis and the accompanying risks for non-communicable diseases [1]. Despite the well-known positive effects of physical activity (PA) and the detrimental effects of sedentary behaviour (SB) on physical and mental health, the majority of children do not meet the PA levels recommended by the World Health Organization (WHO)—at least a daily average of 60 min moderate-to-vigorous PA (MVPA)[2–5]. Additionally, starting from the age of 7 years, children become less active as they grow into adolescents, which may have a negative impact on their growth and maturation [6, 7]. The WHO recommends children limit the time spent in SB, particularly recreational screen time [3]. According to the guiding objectives of the European Workplan on Sport 2021–2024, there is an urgent need to put forth evidence-based policies to promote participation in sports and health-enhancing PA among children to combat the low levels of PA and high levels of SB [8, 9]. However, the factors behind children’s PA/SB engagement are still not sufficiently understood and translated well enough to inform policy makers [10, 11]. To inform policy and implementation of interventions in the real world, a first step is to identify modifiable determinants of PA/SB and prioritize the most effective ones to target [8, 12, 13]. Furthermore, since modifiable determinants may be linked to specific settings (e.g. home, school etc.), the settings of interventions must be considered when assessing their effect [8, 14]. Previous reviews identifying determinants and correlates of PA/SB highlighted issues related to overall low methodological quality in the measurement of outcomes, methodological variability relating to high levels of heterogeneity among studies, and the reliance mostly on self-report measures of PA/SB [15–18]. By nature, PA/SB are diverse behaviours and are influenced by many types of context dependent determinants [8, 10–12].

A large body of research has explored the correlates of PA/SB in children, mainly examining their associations in cross-sectional designs [16–19]. However, to identify modifiable determinants of PA/SB for real-world interventions and policy, the causality between identified determinants and PA/SB, and the modifiability of the determinants need to be examined [8, 10, 11, 20, 21]. Prospective experimental designs, including randomized controlled trials (RCTs) and controlled trials (CTs), can test for causal associations between PA/SB and their determinants. Specifically, RCTs and CTs allow verification of the causal associations by testing manipulated exposures (determinants) and evaluating the effects on PA/SB while controlling for lack of exposure [11, 22]. As such, RCTs and CTs allow both testing of the level of modifiability of determinants and provision of robust evidence for causality between manipulation of determinants and change in PA/SB [8, 22]. The goal of the current review was to contribute to a Best-Evidence Statement (BESt) aimed at informing public policies and interventions based on high-quality evidence regarding the key modifiable determinants of PA/SB in specific settings that should be prioritized and targeted [23].

The settings in which interventions are conducted have received much attention in recent years [14, 24, 25]. To what extent a determinant can be modified to achieve change in PA/SB is highly dependent on the setting of the intervention. Settings refer to social and geographical contexts, which can either be adapted to facilitate change in PA/SB, acting as a modifiable determinant itself, or facilitate modifiability of other determinants to achieve change in PA/SB [14]. A settings-based approach can therefore help identify which determinants underpin PA/SB and unravel how multiple determinants may interact to influence PA/SB [24]. Determinants of PA/SB can be further contextualized from a social-ecological perspective [26]. Using the social-ecological model as a classification framework of determinants can help policymakers target them within their respective domains and settings (e.g., through school/organization) and thereby increase the likelihood of effective interventions. As such, the current review addresses the calls for a settings-based approach to identifying (modifiable) determinants of PA/SB to inform policy, both on intra-/interpersonal levels and societal/policy levels [8, 14, 21, 27].

Moreover, the methods of measurement of PA/SB (e.g. device-based and self-report measures) need to be considered. Different methods of measurement of PA/SB may not yield comparable results, which adds to methodological variability and inconsistency in results [28–30]. For example, both device-based and self-report measures of PA have been found reliable and valid in children, but they do not measure the same aspects of PA [28, 31]. Studies comparing self-report and device-based methods for the measurement of PA in children have shown that self-report measures overestimate PA levels, particularly at higher intensities [30–32]. Therefore, as device-based measures involve continuous accelerometer-based PA/SB tracking, they may be particularly useful in children as they do not rely on recall [30]. Additionally, focussing only on device-based measurement of PA/SB can help mitigate methodological variability, which can contribute to inconsistency in results.

Due to the complexity of PA/SB interventions, few reviews on the determinants (or correlates) of PA/SB in children have conducted meta-analyses, leaving a gap concerning the effectiveness of interventions on determinants of PA/SB [15, 33]. Additionally, previous meta-analyses have mainly focussed on one specific setting (e.g. school or family) and, to the authors’ knowledge, none to date have provided a comprehensive overview on the effectiveness of interventions across settings [33, 34]. Finally, the current review used Bayesian meta-analysis, which provides nuanced conclusions and realistic measures of heterogeneity and adjusts for publication bias [35]. Therefore, the current systematic review and meta-analysis aimed to identify modifiable determinants of device-measured PA and SB targeted in available intervention studies with RCT and CT designs in children and early adolescents (5–12 years old) and quantify the effects of the interventions within their respective settings on the determinants of PA/SB, and the outcomes PA and SB. More specifically, the aim was three-fold: (1) identify determinants of PA and SB targeted in existing interventions, and quantify the effects of the interventions (2) on the identified determinants and (3) on PA and SB.

## Methods

The current systematic review and meta-analysis was part of a series of reviews investigating the effect of interventions on PA/SB and modifiable determinants of PA/SB, with a common search strategy. The methods for the review process were outlined in a pre-published protocol [23]. The review was pre-registered in the International Prospective Register of Systematic Reviews (PROSPERO; 12/10/2021; CRD42021282874). The reporting in the current systematic review and meta-analysis was guided by the Preferred Reporting Items for Systematic Review and Meta-Analysis (PRISMA) [36]. The main outcome measures were device-based PA/SB [3] and modifiable determinants of PA/SB. Based on the WHO definitions, PA was defined as “Any bodily movement produced by skeletal muscles that requires energy expenditure” and SB was defined as “Any waking behaviour characterized by an energy expenditure of 1.5 METs or lower while sitting, reclining or lying” [3]. Modifiable determinants were identified by the context of each intervention, in which factors targeted for manipulation were hypothesized to have an effect on PA/SB [23].

A literature search was conducted in MEDLINE (Ovid), PsycINFO (EBSCO), Web of Science, SPORTDiscus and Cochrane Central Register of Controlled Trials (CENTRAL) on 12/09/2021 (see Table 1 for the search strategy). The search results were filtered for studies published from 2010, when the first global WHO PA guidelines were published, until September 2021, when the initial search was completed. An updated search was conducted on 12/07/2023 to identify studies published after September 2021 following the procedures outlined by Bramer and Bain [37]. The included studies were selected based on study design (RCTs and CTs only), measurement method for PA/SB (device-based only) and population (children and adolescents).

**Table 1:** Search strategy including Boolean operators for each domain

Domain	Search terms
Outcome: Physical activity behaviour^a^	("Physical activ*") OR (exercise) OR (sport*) OR (play) OR (exertion) OR (recreation) OR (training) OR ("motor activit*") OR ("physical performance") OR ("physical movement") OR ("physical effort") OR (exergaming)
**OR**	
Outcome: Sedentary behaviour^a^	(sedentar*) OR ("screen time") OR (gaming) OR ("computer use") OR (sitting) OR (inactiv*) OR ("seated posture") OR ((watch* or view*) N/2 (TV or television))
**AND**	
Target population^a^	(child*) OR (youth) OR (adolescen*) OR ("young people") OR ("school age*") OR (p?ediatric) OR (juvenile) OR (teen*)
**AND**	
Study design^b^	(RCT) OR ("control* trial*") OR (quasi) OR (longitudinal) OR (intervention*) OR (prospective) OR ("follow up")
**OR**	
Determinants^b^	(determinant*) OR (antecedent*) OR (predictor*) OR (mediator*) OR (moderator*) OR (exposure*)
**AND**	
Measurement methods^b^	(acceleromet*) OR ("activity profile") OR (recall) OR (diary) OR ("activity monitor*") OR ("heart rate monitor*") OR ("direct observation") OR (Actigraph*) OR ("activity track*") OR ("self report*") OR (survey) OR (pedomet*) OR (wearable*)

^a^Restricted search to title, abstract and keywords

^b^Search in entire study

The screening, data extraction and risk of bias assessment were completed by a group of 26 reviewers, who were experienced researchers. The records resulting from the search were screened initially by one member of the review team to remove duplicates and any grey literature. The screening of each record by title and abstract and by full-text was completed in Covidence [38] by two blinded reviewers, with one additional reviewer to resolve any conflicts. Each reviewer screened approximately 2000 records at the title and abstract stage and ca 70 records at the full-text stage. A pre-piloted decision tree based on the inclusion and exclusion criteria was used for the screening to promote consistency among reviewers. Studies were included if they comprised a sample of children and/or early adolescents with a mean age within the range of 5.00–12.99 years (henceforth termed children), an RCT or CT study design, a device-based measure of PA and/or SB, modifiable determinants, measures of the outcomes included at least at two timepoints (pre- and post-intervention), and a control group. Studies were excluded if they included non-clinical populations, i.e. participants with diagnosed medical conditions known to affect the ability to engage in PA and/or patients undergoing treatment on all levels of care (e.g. studies including patients with cancer or individuals with anterior cruciate ligament injury or studies where the intervention takes place in a clinical setting). Studies were also excluded if they included participants with disabilities (i.e. impairments, activity limitations and participation restrictions, denoting the negative aspects of the interaction between an individual and that individual’s contextual factors). Additionally, studies published in languages other than English for which translation could not be obtained were excluded. Data extraction and risk of bias assessment were also completed in Covidence [38] by two independent reviewers, each completing approximately six records. Reviewers discussed any conflicts to resolve them, and a third reviewer cross-checked and resolved any remaining conflicts in both data extraction and risk of bias assessment. The overall characteristics of the included studies and numerical data for the outcomes for all time points were extracted for use in meta-analyses. Authors were contacted to supplement additional or unreported data. The extracted data included intervention description (design, intervention content, control activity, location), sample description (sample size, grouping, sex, age), outcome measures (targeted determinants, type of PA/SB measures, instruments used to measure outcomes), time frames (duration of intervention, time between measures and follow-up) and quantitative outcome data (measures of central tendency and variance for each outcome measure).

Modified versions of Cochrane’s tools for risk of bias tools were used—Risk of Bias in Non-randomised Studies of Interventions (ROBINS-I) for CTs [39] and Cochrane Risk of Bias Tool for Randomised Trials (RoB2) for RCTs [40]. The modified risk of bias tools included an additional domain each to assess the risk of bias in the measurement of the determinant(s). The quality of the produced evidence in each meta-analysis was assessed using the Grading of Recommendations Assessment, Development and Evaluation (GRADE) approach in GRADEpro [41]. The GRADE assessment considers the results of the meta-analysis, their inconsistency, indirectness and imprecision, and the overall risk of bias of the included studies and yields a score on the quality of evidence provided by the meta-analysis.

### Data Synthesis

The extracted data of the study characteristics are summarized and discussed narratively. The identified modifiable determinants were categorized according to the domains of the social-ecological model and listed along with the study characteristics. Levels of the social-ecological model as applied in the current review include individual (relating to psychological, physiological and behavioural determinants, e.g. self-efficacy, aerobic/anaerobic capacity, diet), interpersonal (relating to behaviours when interacting with others, e.g. social support, co-PA) and policy environment (relating to physical environment and organizational policy, e.g. perceived physical environment, provision of PA spaces) levels [26].

The quantitative data extracted from the included studies were continuous. Effect sizes (Cohen’s *d*) were calculated, with the standard error (SE) and 95% confidence intervals (95% CI). Mean and standard deviation (SD) were used to calculate the effect sizes. For studies reporting other measures of central tendency or variance (e.g. median and interquartile range) and for studies reporting change from baseline (e.g. mean change and 95% CI for the change), mean and SD were calculated for those [42–45]. For studies reporting more than one intervention group targeting different outcomes, the intervention groups with the relevant outcomes were included (e.g. for studies reporting both PA and eating behaviour, PA was included) [46]. For studies reporting more than one intervention group with the same outcome, combined scores were calculated for the intervention groups and the sample sizes for the intervention groups were summed to avoid duplicate entries of the shared control groups [46, 47]. Combined scores were calculated for some conceptually similar determinants to provide a broader definition in line with theoretical convention (e.g. a combined score of intrinsic and identified motivation to yield autonomous motivation) [48].

Meta-analyses were conducted in JASP (version 0.17.1) [49], using the Bayesian statistical approach. Classical meta-analyses (based on frequentist statistical inference) were also conducted (see Supplementary file S1 for details on methodology and Tables S1-S2 for the results). The Bayesian approach has some advantages. First, it allows inclusion of prior data into a meta-analysis to update existing knowledge, providing a cumulative indication of effects. Second, more nuanced conclusions can be drawn based on the probability that the null or alternative hypothesis is true, rather than adopting a dichotomous method that determines support for the alternative hypothesis solely based on a p-value. Third, Bayesian meta-analysis can provide more realistic measures of heterogeneity and publication bias as they are modelled explicitly, which makes it possible to account for them [50, 51]. Meta-analyses were conducted to investigate the post-intervention effect (immediately after intervention ceased), short-term maintenance effect (using follow-up measured ≤ 6 months post-intervention) and long-term maintenance effect (using follow-up measured > 6 months post-intervention) for the PA and SB outcomes and the determinants. Within each setting, data were pooled to conduct meta-analyses for each determinant, as well as for the PA and SB outcomes. As such, results were reported for the intervention effects on each determinant, and for PA and SB separately. For the PA and SB outcomes, separate analyses were conducted for measurements that represented whole-day PA/SB (including all waking hours) and measurements that represented part-day PA/SB (e.g. only during physical education or active transport). For studies reporting both PA and SB, both were included in the respective meta-analyses. Finally, the analyses were conducted separately for CTs and RCTs to account for differences in level of evidence that each study design generates.

Robust Bayesian meta-analysis (RoBMA) with Markov Chain Montecarlo (MCMC) estimation was used to conduct publication bias-adjusted meta-analyses [35, 52]. Publication bias is an estimate of the bias in effect sizes due to preferential publishing of statistically significant results in studies while retaining non-significant ones that are essential to cumulative meta-analyses. As such, RobMA provides publication bias-adjusted effect sizes that allow for a more objective interpretation of effect sizes without common overestimations due to publication bias with unknown magnitude [35]. To enable random effects models, conditional estimates were used with the prior specifications and models, with the modification of removing the fixed-effects models [52]. Mean effect with 95% credible interval (95% CrI), Bayes factor 10 (BF_10_) for the effect (ES BF_10_), mean heterogeneity (*τ*) with 95% CrI and publication bias (PB BF_10_) were reported. For the interpretation of ES BF_10_ and PB BF_10_, the following benchmarks were used: > 100 extreme evidence, 30–100 very strong, 10–30 strong evidence, 3–10 moderate evidence, 1–3 trivial evidence, 1 no evidence, 1/3–1 trivial evidence, 1/10–1/3 moderate evidence, 1/30–1/10 strong evidence, 1/100–1/30 very strong evidence, and < 1/100 extreme evidence [53].

Finally, as mentioned in the pre-published protocol [23], analyses to investigate the links between the effects on determinants and PA/SB (such as meta-analytic structural equation modelling) were planned to provide a stronger basis for a causal relationship between the modifiable determinants and PA/SB. However, data were not available in the included studies to conduct such analyses.

## Results

### Study Selection and Characteristics

**Fig. 1:**
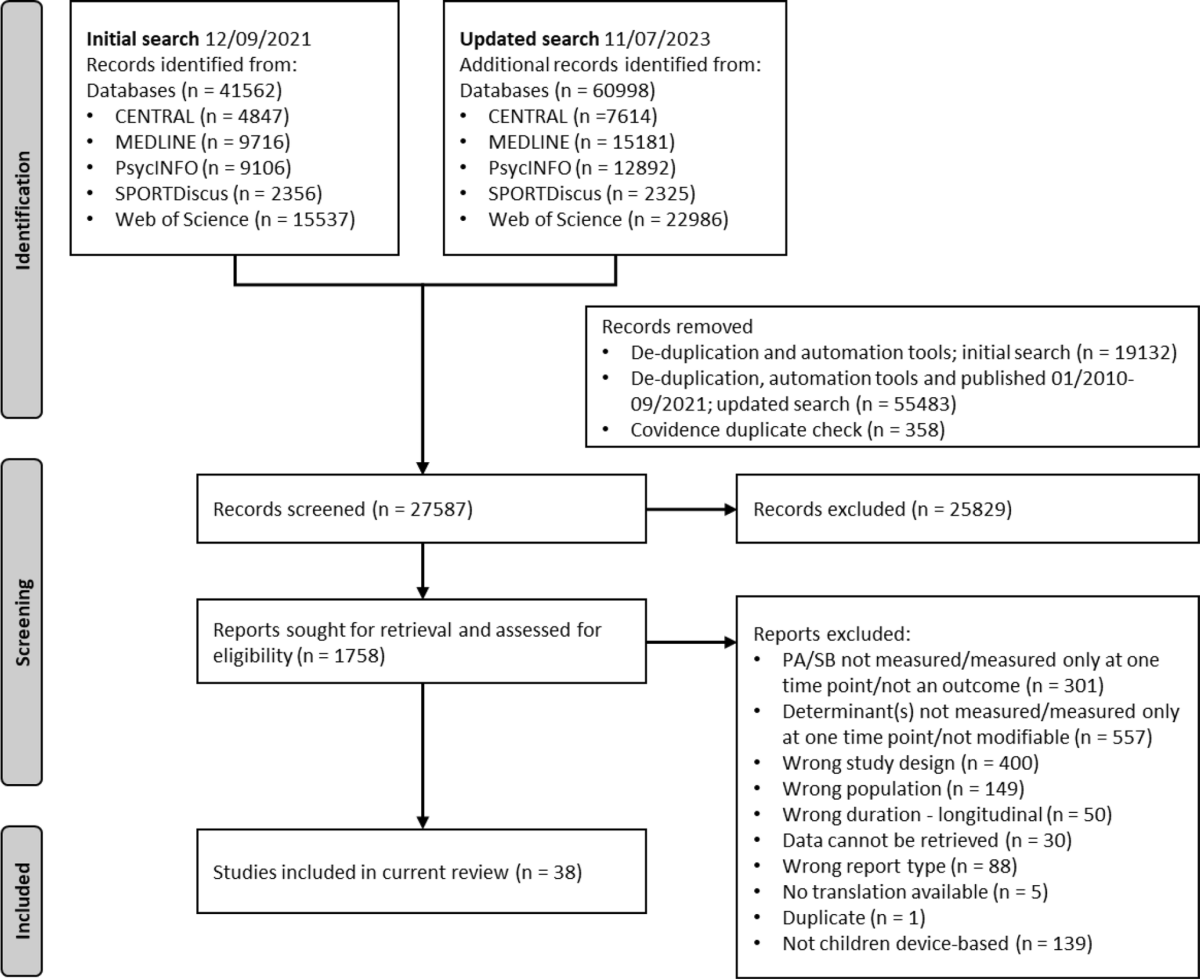
PRISMA flow diagram outlining screening and selection procedure

The literature searches yielded 41,562 records for the initial search and 60,998 records (i.e., 19,436 additional records) for the updated search, of which 27,587 titles and abstracts and subsequently 1758 full texts were screened. Finally, 38 studies targeting children (5–12 years old) and reporting device-based measures of PA and/or SB were included in the current review (Fig. 1).

The included studies (summarized and numbered in Table 2) consisted of 29 RCTs, including 10 cluster-RCTs and 9 CTs. Three settings and combinations of these were identified, including school (*n* = 24), family/home (*n* = 8) and community (*n* = 1), a combination of school and family/home (*n* = 4), and a combination of community and family/home (*n* = 1). The content of the interventions and their aims are summarized in Table 2. Out of the 38 studies, 15 included measures of both PA and SB, 22 included only a measure of PA and two included only a measure of SB. In studies including measures of both PA and SB, no distinctions were made for whether the determinants were of PA or SB. Of the 38 included studies, 31 reported measures of whole-day PA/SB and 7 reported measures of part-day PA/SB. Follow-up measurements were reported in 11 studies, while seven studies reported short-term follow-up measures (≤ 6 months post-intervention) and 4 studies reported long-term follow-up measures (> 6 months post-intervention). The total sample size in the included studies was *n* = 14,258 and ranged between 29 and 3147 participants. Girls made up 67% (*n* = 9548) of the total sample, with seven studies including 100% girls. Theories or combinations of theories were used as the basis for the interventions in 30 studies (79%), with eight studies not reporting any theory/theoretical framework.

**Table 2:** Study characteristics

Study	Study aim	Study design country	Age (years) *N* (by sex, by group)	Intervention content	Setting	Determinants (category)	Outcome measure method	Measurement timepoints	Theoretical framework
Alhassan et al., 2018 [54]	To examine the feasibility and efficacy of a 12-week culturally tailored mother-daughter PA intervention on the PA levels of pre-adolescent African-American girls	RCT USA	8.3 (1.3) *n* = 76 Girls *n* = 76 (100%) Boys *n* = 0 CH *n* = 25 CH-M *n* = 28 Control *n* = 23	3 × /week for 12 weeks: 2 h tutoring and snacks; 1h dance class; Weekly health newsletter CH-M attended by mothers and daughters; CH attended by daughters only CON: received intervention later	School Family/home	Parental PA behaviour (EBH) Self-efficacy (IPS) PA preference (IPS)	Daily MVPA (%) Daily SB (%) Actigraph GT1M, GT3X	T1: baseline T2: 6 weeks (mid) T3: 3 months (post)	Social cognitive theory
Barnes et al., 2015 [55]	To evaluate the feasibility and preliminary efficacy of a mother-daughter program targeting improvements in PA levels	RCT Australia	8.5 (1.7) *n* = 48 Girls *n* = 48 (100%) Boys* n* = 0 MADE4Life *n* = 25 Control *n* = 23	1 × /weeks for 8 weeks: mothers and daughters attended 25 min separate education sessions and 60 min PA sessions together CON: received intervention later	Family/home	Social support—parents (ESS) Parental PA behaviour (EBH) Parenting for PA (EBH)	Daily PA Mean (CPM) Daily SB (%) Actigraph GT3X +	T1: baseline T2: 10 weeks (post) T3: 3 months (follow-up)	Social cognitive theory
Bergh et al., 2012 [56]	To examine whether changes in personal, social and physical-environmental determinants mediated the effect of change in PA behaviour in the ‘Health In Adolescents’ study	c-RCT Norway	11.2 (0.3) *n* = 700 Girls *n* = 392 (56%) Boys *n* = 308 Intervention *n* = 215 Control *n* = 485	Increasing PA opportunities Children: lessons with information on PA, access to equipment during recess, active commuting campaigns, active breaks Parents: information sheets Teachers: inspirational courses and resources CON: no intervention	School	Self-efficacy (IPS) Enjoyment (IPS) Social support—parents (ESS) Social support—friends (ESS) Social support—teachers (ESS) Perception of physical environment (PEN)	Daily PA Mean (CPM) Actigraph GT1M/CSA	T1: baseline T2: 20 months (post)	Social cognitive theory
Breslin et al., 2019 [57]	To determine whether a theory-driven multicomponent ‘Sport for LIFE: All Island’ programme improved PA, health-related quality of life and nutritional attitudes and behaviours in 8–9-year-old children from low socioeconomic status across Ireland	c-RCT Ireland	8.7 (0.5) *n* = 740 Girls *n* = 363 (49%) Boys *n* = 377 Intervention *n* = 383 Control *n* = 357	1 × /week for 12 weeks: education sessions for children in weekly themes on PA benefits and nutrition; intervention ended with PA festival targeting long-term goals inspiration CON: received intervention later	School	Social support—friends (ESS) Moods and emotions (IPS) Physical well-being (IPS) Psychological well-being (IPS) Parental relations and autonomy (EBH)	Daily MVPA (CPM) Actigraph GT3X	T1: baseline T2: 6 weeks (mid) T3: 13 weeks (post) T4: 6 months (follow-up)	Social cognitive theory
Carlin et al., 2018 [58]	To investigate the feasibility of peer-led brisk ‘Walking In ScHools’ intervention (the WISH study) and to investigate the impact of participating in a 12-week school-based walking programme on school-time PA and sedentary behaviour post-intervention (week 12) and at follow-up (6 months)	c-RCT UK	12.4 (0.6) *n* = 199 Girls *n* = 199 (100%) Boys *n* = 0 Intervention *n* = 101 Control *n* = 98	3 × /day for 12 weeks: structured walking sessions. Led by older pupils at the school (15–17 years) who received training	School	Self-efficacy (IPS) Social support—parents (ESS) Social support –friends (ESS) Benefits of PA (IPS) Barriers to PA (IPS)	School time PA (min/school day) School time SB (min/day) Actigraph GT3	T1: baseline T2: 12 weeks (post) T3: 6 months (follow-up)	Social cognitive theory
Carson et al., 2013 [59]	Assessing whether changes have occurred in targeted mediators as well as in outcome behaviours at mid-intervention can help determine whether the study is proceeding as planned	c-RCT Australia	8.0 (1.3) *n* = 293 Girls *n* = 163 (56%) Boys *n* = 130 Increase PA *n* = 75 Reduce SB *n* = 74 Increase PA + Reduce SB *n* = 80 Control *n* = 64	Reduce SB: one standing class of 30 min every day, 2-min activity break every 30 min. Parents received newsletters with key learning messages Increase PA: promotion of PA at lunch and recess, provision of equipment, signage, and line markings on grounds Reduce SB + increase PA: combination of interventions increase PA + reduce SB	School	Enjoyment (IPS) Perception of physical environment (PEN) Parenting for PA (EBH)	Daily sedentary time (min/day) Actigraph Model GT3X	T1: baseline T2: 5–9 months (post)	Social cognitive theory Behavioural choice theory Ecological systems theory
Chen et al., 2011 [60]	To examine the efficacy of the Web-Based Active Balance Childhood program in promoting healthy lifestyles and healthy weight in Chinese American adolescents	RCT USA	12.5 (3.2) *n* = 54 Girls *n* = 29 (53%) Boys *n* = 25 Intervention *n* = 26 Control *n* = 25	1 × /week for 8 weeks: PA and nutrition lesson for participants Total 3 lessons for parents Intervention adapted to behavioural stage of participants	Family/home	Self-efficacy (IPS) PA knowledge (IPS)	Daily MVPA (CPM) Actigraph MTI/CSA	T1: baseline T2: 8 weeks (post) T3: 6 months (for control)/8 months (for Intervention) (follow-up)	Transtheoretical model—stages of change and social cognitive theory
Cliff et al., 2011 [61]	To examine the impact of a competence motivation theory framed PA intervention targeting movement skill development	RCT Australia	8.3 (1.0) *n* = 165 Girls *n* = 97 (59%) Boys *n* = 68 PA* n* = 63 Diet *n* = 42 PA + diet* n* = 60 Diet used as control PA + diet excluded	Phase 1: 1 × /week for 10 weeks: 2-h session including 90-min PA, improving fundamental movement skills and fostering success experience; home challenges involving parents; parent workshop at the end of intervention Activities adapted to movement skill development level of participants Phase 2: 1 × /month for 3 months: follow-up phone calls; 1 × movement skill booster session	Community Family/home	Motor competence (IPH) Perceived athletic competence (IPH)	Daily MVPA (CPM) Actigraph 7164	T1: baseline T2: 6 months (post) T3: 12 months (follow-up)	Competence motivation theory
Cohen et al., 2015 [62]	To evaluate the effects of the ‘Supporting Children’s Outcomes using Rewards, Exercise, and Skills’ (SCORES) program, a 12-month school-based c-RCT designed to increase PA and improve fundamental movement skills competency among children attending primary schools in low-income communities	c-RCT Australia	8.5 (0.6) *n* = 460 Girls *n* = 248 (54%) Boys *n* = 212 Intervention *n* = 199 Control *n* = 261	12-month intervention: Phase 1: teacher professional learning, student leadership workshops, and PA promotion tasks Phase 2: address school PA policies Phase 3: address strategies to improve school-community links with local organizations to assist with PA-related programs	School	Motor competence (IPH)	Total daily PA (CPM) Actigraph GT3X +	T1: Baseline T2: 6 months (mid) T3: 12 months (post)	Socioecological model
Cohen et al., 2017 [63]	To examine whether changes in individual (i.e. enjoyment and perceived competence), social (i.e. social support from teachers, parents and peers) and environmental (i.e., access to PA facilities, equipment, and opportunities in the local community) constructs from the theoretical models mediated PA changes in the SCORES intervention	c-RCT Australia	8.5 (0.6) *n* = 460 Girls *n* = 248 (54%) Boys *n* = 212 Intervention *n* = 199 Control *n* = 262	12-month intervention: Phase 1: teacher professional learning, student leadership workshops, and PA promotion tasks Phase 2: address school PA policies Phase 3: address strategies to improve school-community links with local organizations to assist with PA-related programs	School	Enjoyment (IPS) Social support—parents (ESS) Social support—friends (ESS) Social support—teachers (ESS) Perceived athletic competence (IPH)	Total daily PA (CPM) Actigraph GT3X +	T1: Baseline T2: 12 months (post)	Socioecological model
Comeras-Chueca et al., 2022 [64]	To examine the influence of an AVG intervention combined with multicomponent exercise on muscular fitness, PA, and motor skills in children with overweight or obesity	RCT Spain	10.1 (0.8) *n* = 29 Girls *n* = 13 (45%) Boys *n* = 16 AVG *n* = 21 Control *n* = 8	3 × /week for 5 months: 60 min active video games combined with multi-component exercise—both with progression in difficulty and intensity	Community	Motor competence (IPH) Muscular fitness (IPH)	Total PA (min/day) GENEActiv accelerometer	T1: Baseline T2: 5 months (post)	NA
Duck et al., 2021 [65]	To examine the feasibility and preliminary effectiveness of using wearable activity tracker technology, integrated with altruistic motivation in children to increase PA, fitness, and prosocial behaviour	CT USA	9.2 (0.4) *n* = 35 Girls *n* = 23 (66%) Boys *n* = 12 Intervention *n* = 17 Control *n* = 18	For 10 weeks: children track PA levels and earn PA points that could be used to provide food to undernourished children internationally	School	Prosocial behaviour (IBH)	Daily MVPA (min/day) Actigraph GT3X	T1: baseline T2: 10 weeks (post)	NA
Eather et al., 2013 [66]	To explore hypothesized mediators of PA behaviour change in the ‘Fit-4-Fun’ group RCT	c-RCT Australia	10.7 (0.6) *n* = 226 Girls *n* = 118 (52%) Boys *n* = 108 Intervention *n* = 118 Control *n* = 108	1 × /week for 8 weeks: 60-min health and physical education sessions with aim to understand PA-related benefits 3 × /week for 8 weeks: Family—20-min home activities aiming to improve home-based PA with focus on fitness and flexibility School: encourage PA participation during recess with provision of activity/game cards and equipment, led by students CON: usual health physical education lessons. Received intervention later	School Family/home	Enjoyment (IPS) Self-efficacy (IPS) Social support—parents (ESS) Social support—friends (ESS) Perception of physical environment (PEN)	Daily (step count (steps/day) Yamax pedometer	T1: baseline T2: 3 months (post) T3: 6 months (follow-up)	Social cognitive theory competence motivation theory
Fu et al., 2013 [67]	To examine the effects of a health-related physical fitness-based basketball program on middle school students’ in-class PA, perceived competence, and enjoyment as compared to the effects on those study variables shown by a control group participating in the traditional approach basketball unit	CT USA	12.6 (0.6) *n* = 61 Girls *n* = 36 (59%) Boys *n* = 25 Intervention *n* = 31 Control *n* = 20	1 × /week for 6 weeks: 50-min health-related physical fitness basketball classes. Structure and progression focussed on building physical/aerobic fitness and movement skills CON: 1 × /week for 6 weeks: 50- min traditional basketball sessions with not particular progression or focus	School	Perceived athletic competence (IPH) Enjoyment (IPS)	In-class PA (steps/min) Piezoelectric pedometer	T1: baseline T2: 6 weeks (post)	NA
Gao et al., 2019 [68]	To investigate the effects of a 9-month school-based active video game intervention on the energy expenditure and psychosocial beliefs of children from low-socioeconomic status families living in underserved urban areas in Minnesota	CT USA	9.2 (0.6) *n* = 81 Girls *n* = 39 (48%) Boys *n* = 42 Intervention *n* = 36 Control *n* = 45	1 × /week for 9 months: 50-min session of active video games CON: no active video games	School	Self-efficacy (IPS) Social support—general (ESS) PA outcome expectancies (IPS)	Daily Energy expenditure (METs) Actigraph GT3X +	T1: baseline T2: 4 months (mid) T3: 9 months (post)	Social cognitive theory
Gu et al., 2018 [69]	To examine the impact of types of pedometer-based goal setting on children’s motivation, motor performance and PA in physical education	RCT USA	10.9 (0.8) *n* = 273 Girls *n* = 137 (50%) Boys *n* = 136 ISG *n* = 110 GSG *n* = 90 Control *n* = 73	Three physical education classes/week for 8 weeks: Children were given a weekly step count target ISG: personalized step count target based on baseline measure with a progression when reaching target GSG: fixed step count target CON: usual PE without pedometer	School	Motor competence (IPH) PA outcome expectancies (IPS)	PA during PE (steps/class) Pedometer	T1: baseline T2: 8 weeks (post)	Goal-setting theory
Hamilton et al., 2020 [70]	To determine if a school-based video intervention, designed and implemented using a community-based participatory research approach, improved daily MVPA through improving the psychosocial constructs (self-efficacy, knowledge, skills, attitudes, and beliefs) associated with increased PA of minority children residing in a rural community	RCT USA	10.3 (0.5) *n* = 39 Girls* n* = 19 (48%) Boys *n* = 20 Intervention *n* = 20 Control *n* = 19	2 × /week for 4 weeks: first 15 min of PE viewing videos addressing psychosocial constructs to lead to increased PA, e.g. PA goal setting, enjoyable PA, PA, barriers, self-talk, benefits of PA, types of PA CON: usual PE, no videos	School	Self-efficacy (IPS) PA knowledge (IPS) Psychosocial skills (IPS)	Daily MVPA (min/day) Actigraph wGT3X-BT	T1: baseline T2: 4 weeks (post)	Social cognitive theory transtheoretical model
Harrington et al., 2018 [71]	To assess the effectiveness of the ‘Girls Active’ PA programme in UK secondary schools	RCT UK	12.8 (0.8) *n* = 1752 Girls *n* = 1752 (100%) Boys *n* = 0 Control *n* = 885 Intervention *n* = 867	14-month intervention Self-review of school PA, PE and sport culture by teachers and school leadership. Preparation of material and strategies for PA promotion by teachers/school. Peer leadership group of girls 11–14 years to engage with school staff on PA policymaking, PA promotion	School	Amotivation (IPS) Attitudes (IPS) Autonomous motivation (IPS) Controlled motivation (IPS) Enjoyment (IPS) Intentions (IPS) PA outcome expectancies (IPS) Perception of physical environment (PEN) Physical self-perceptions (IPS) Self-efficacy (IPS) Social support—Friends (ESS) Social support—Parents (ESS) Social support—Teachers (ESS)	Daily MVPA (min/day) Daily SB (min/day) GENEActiv accelerometer	T1: baseline T2: 7 months (mid) T3: 14 months (post)	Social cognitive theory
Johnstone et al., 2017 [73]	To determine if participation in the ‘Go2Play Active Play’ intervention improved (a) school day PA and (b) fundamental movement skills	CT UK	7.0 (1.0) *n* = 196 Girls *n* = 90 (46%) Boys *n* = 106 Intervention *n* = 63 Control *n* = 18	1 × /week for 10 weeks: 1-h active play session to improve physical literacy facilitated by play workers; 30 min facilitated games, 30 min free play CON: usual PE classes, no play intervention	School	Motor competence (IPH)	School-day PA (CPM) School-day SB (%) Actigraph GT3X	T1: baseline T2: 5 months (post)	NA
Johnstone et al., 2019 [72]	To determine the feasibility of an active play intervention to inform a future definitive RCT	c-RCT UK	7.1 (0.3) *n* = 137 Girls *n* = 79 (58%) Boys *n* = 58 Intervention *n* = 73 Control *n* = 64	1 × /week for 10 weeks: 1-h active play session to improve physical literacy facilitated by play workers; 30 min facilitated games, 30 min free play CON: usual PE classes, no play intervention	School	Motor competence (IPH)	School time PA (%) Actigraph GT3X	T1: baseline T2: 9 weeks (post)	NA
Latomme et al., 2023 [91]	To evaluate the efficacy of a newly developed lifestyle intervention for fathers and their children, ‘Run Daddy Run’, on objectively measured PA of fathers and their children, by increasing co-PA. We hypothesized that in the intervention group (compared to the control group), father-child co-PA and fathers’ and children’s PA will significantly improve	CT Netherlands	7.1 (0.9) *n* = 98 Girls *n* = 57 (58%) Boys *n* = 41 Intervention* n* = 35 Control *n* = 63	6 × 120 min (bi-weekly): interactive sessions for fathers and children. Including education and practical elements eHealth component online: setting weekly co-PA goals and tracking co-PA. Exercise/activity library Father support chat group CON: no intervention	Family/home	Co-PA (EBH) Parental PA behaviour (EBH) Parenting for PA (EBH) Family health climate (ESS)	Total-day PA Total-day SB Axivity AX3, 3-axial	T1: baseline T2: 14 weeks	Behaviour change wheel
Laukkanen et al., 2017 [74]	To examine whether a family-based PA intervention, consisting of individually tailored face-to-face and phone counselling for parents of children, influenced parental support of children’s PA and objectively measured leisure-time PA in children with the lowest and highest initial parental support	c-RCT Finland	6.1 (1.2) *n* = 91 Girls *n* = 47 (52%) Boys *n* = 44 Intervention *n* = 44 Control *n* = 47	Over 6 months: nine behaviour change techniques employed in a lecture, individual face-to-face counselling, goal setting, phone consultation: providing instruction, providing information on consequences, prompting identification as a role model, providing general encouragement, providing information about others’ approval, prompting intention formation, progressive goal setting, prompting barrier identification and self-evaluation	Family/home	Social support—parents (ESS)	Total MVPA (min/day) Total SB (min/day) Triaxial X6-1a accelerometer	T1: baseline T2: 6 months (post) T3: 12 months (follow-up)	Social cognitive theory Theory of planned behaviour
Lee and Gao, 2020 [75]	To investigate whether teacher-managed app-integration (e.g. Educreations, Garage Band, Timer, Team Shake) via a tablet could improve children’s beliefs and increase their PA levels in PE	CT USA	9.7 (0.6) *n* = 157 Girls *n* = 73 (47%) Boys *n* = 84 App-enhanced *n* = 77 Control *n* = 80)	5 × /week for 6 weeks: 50-min app-integrated PE classes. Apps used to help activities taught during the classes, providing timing, instructions, visual feedback and encouragement for engagement	School	Self-efficacy (IPS) Enjoyment (IPS) Social support—general (ESS) PA outcome expectancies (IPS)	In-session MVPA (%/min) In-session SB (%/min) Actigraph GT3X +	T1: baseline T2: 6 weeks (post)	Social cognitive theory
Lloyd et al., 2015 [76]	To examine the mediators of children’s PA and dietary behavior change in the ‘Healthy Dads Healthy Kids’ community RCT	RCT Australia	8.7 (2.3) *n* = 45 Girls *n* = 18 (40%) Boys *n* = 27 HDHK *n* = 23 Control *n* = 22	1 × /week for 7 weeks: four fathers-only theoretical 90-min sessions; three practical father and child sessions. Encourage father-child time, co-PA and modelling of health behaviours Fathers received resources: handbook of completed sessions, logbook for self-monitoring, list of home-based tasks. Children received a kids-handbook for home-based activities CON: wait-list control	Family/home	Co-PA (EBH) Parental PA modelling (ESS) Self-efficacy (IPS) PA outcome expectancies (IPS) Social support—parents (ESS) Parenting for PA (EBH)	Daily (step count (steps/day) Pedometer	T1: baseline T2: 12 weeks (post)	Family systems theory social cognitive theory
Lonsdale et al., 2019 [77]	To test the efficacy of a teacher professional learning intervention, delivered partially via the internet, designed to maximize opportunities for students to be active during physical education lessons and enhance adolescents’ motivation towards physical education and PA	c-RCT Australia	12.9 (0.6) *n* = 1421 Girls *n* = 796 (56%) Boys *n* = 625 Intervention *n* = 693 Control *n* = 728	Workshop given by research staff to teachers with tasks on project website, including self-reflection and strategy for implementation, and mentoring by research staff Intervention targeted teachers to deliver lessons that maximized opportunities for MVPA and enhance their students’ motivation towards PE	School	Social support—teachers (ESS) Amotivation (IPS) Controlled motivation (IPS) Autonomous motivation (IPS)	PE-lesson MVPA (CPM) PE-lesson SB (CPM) Actigraph GT1M, GT3X, GT3X +	T1: baseline T2: 7–8 months (post) T3: 14–15 months (follow-up)	Self-determination theory
Manley et al., 2014 [78]	To examine the effectiveness of a school-based pedometer intervention program in improving self-efficacy, PA, BMI and aerobic fitness levels of 11–13-year-old children	RCT USA	11.6 (0.7) *n* = 116 Girls *n* = 59 (51%) Boys *n* = 47 HDHK *n* = 55 Control *n* = 61	Daily for 12 weeks: Children provided with pedometer and given a step count goal during school hours: boys 15,000 steps and girls 12,000 steps. Children recorded step count at end of day Daily additional 10 min of PA led by teacher CON: No pedometers. Received intervention later	School	Self-efficacy (IPS) Aerobic fitness (IPH)	Daily (step count (steps/day) Yamax Digiwalker 200 pedometer	T1: baseline T2: 14 weeks (post)	Social cognitive theory
Morgan et al., 2014 [79]	To implement and evaluate the HDHK intervention, when delivered by trained local facilitators	RCT Australia	8.1 (2.1) *n* = 132 Girls *n* = 59 (49%) Boys *n* = 73 HDHK *n* = 60 Control *n* = 72	1 × /week for 7 weeks: four fathers-only theoretical 90-min sessions; three practical father and child sessions. Encourage father-child time, co-PA and modelling of health behaviours Resources: handbook of home-based activities, logbook for self-monitoring, kids-handbook for home-based activities CON: wait-list control	Family/home	Parental PA behaviour (EBH)	Daily (step count (steps/day) SW200 Pedometer	T1: baseline T2: 14 weeks (post)	Social cognitive theory family Systems theory
Morgan et al., 2021 [80]	To implement and evaluate the ‘Dads and Daughters Exercising and Empowered’ intervention when delivered in community settings by local trained facilitators on the PA levels of fathers and daughters and a host of secondary outcomes	RCT Australia New Zealand	8.2 (1.8) *n* = 193 Girls *n* = 193 (100%) Boys *n* = 0 HDHK *n* = 98 Control *n* = 95	1 × /week for 9 weeks: 90-min sessions including a 15-min education session with fathers and daughters, separate, concurrent 30-min education sessions for fathers and daughters and a 45-min practical session for fathers and daughters together. Promoting fathers and daughters PA modelling for each other Resources: father’s logbook, daughter’s booklet, and sport skills booklet, including home-based activities, goal setting, tracking co-PA and skill development CON: wait-list control	Family/home	Motor competence (IPH) Perceived athletic competence (IPH) Co-PA (EBH) Parenting for PA (EBH) Parental PA modelling (ESS)	Daily (step count (steps/day) Pedometer	T1: baseline T2: 12 weeks (post)	Self-determination theory Social cognitive theory
Pearce and Dollman, 2019 [81]	To evaluate whether ‘active play’ in children in grades three to seven in a socially disadvantaged school, particularly in the ‘after-school’ 3 p.m. to 6 p.m. period increased device measured and self-reported PA	CT Australia	Range = 8–13 (no mean) *n* = 147 Girls *n* = 87 (59%) Boys *n* = 60 Intervention *n* = 63 Control *n* = 84	1 × /week for 10 weeks: 60-min sessions, including classroom training 15–20 min and 40–45 min physical activity Resources: Home-based activity booklet 4 × 60-min parent workshops promote awareness of a healthy diet and the importance of exercise	School Family/home	Self-management strategies (IPS) Self-efficacy (IPS) Enjoyment (IPS) Social support—parents (ESS) Social support—teachers (ESS) PA outcome expectancies (IPS) Barriers to PA (IPS)	Daily MVPA (min/day) Actigraph GT3X +	T1: baseline T2: 10 weeks (post) T3: 20 weeks (follow-up)	NA
Rhodes et al., 2021[82]	To examine parents’ perceived family PA frequency (co-PA), family PA social cognitions, and family PA habit over the course of a family planning intervention	RCT Canada	Range = 6–12 9 (No SD) *n* = 102 Girls *n* = 53 (52%) Boys *n* = 49 Intervention *n* = 52 Control *n* = 50	Received Canada’s PA guidelines, workbook how to plan for family PA and practical material for planning family PA CON: received Canada’s PA guidelines	Family/home	Norms (INT) Co-PA (EBH) Parenting for PA (EBH) Automaticity (IPS) Attitudes (IPS) Intentions (IPS) Perceived behavioural control (IPS)	Daily MVPA (min/day) Actigraph GT3X	T1: baseline T2: 6 weeks (mid) T3: 13 weeks (mid) T4: 26 weeks (post)	Theory of planned behaviour
Robbins et al., 2012 [84]	To determine whether girls in one school receiving nurse counselling plus an after-school PA club showed greater improvement in PA, cardiovascular fitness, and body composition than girls assigned to an attention control condition in another school	CT USA	11.4 (0.7) *n* = 69 Girls *n* = 69 (100%) Boys *n* = 69 Intervention *n* = 37 Control *n* = 32	5 × /week for 6 months: 90-min after-school physical activity club, including 60 min PA session, 20 min health discussion, 20 min snack/homework 3 × motivational, individually tailored counselling sessions	School	Aerobic fitness (IPH) Barriers to PA (IPS) Benefits of PA (IPS) Norms (INT) Exposure to PA models (ESS) Social support—general (ESS)	Daily MVPA (min/hour) Actigraph GT1M	T1: baseline T2: 6 months (post)	Health promotion model
Robbins et al., 2019 [83]	To examine whether constructs, including benefits of PA, barriers to PA, PA self-efficacy, social support for PA, enjoyment of PA and motivation, derived from the Health Promotion Model and Self-Determination Theory mediated changes in accelerometer-measured MVPA following a school-based PA intervention for adolescent girls	RCT USA	12.1 (1.0) *n* = 1519 Girls *n* = 1519 (100%) Boys *n* = 0 Intervention* n* = 753 Control *n* = 766	2 × face-to-face motivational interviewing sessions at beginning and end of intervention 3 × /week afterschool club with providing fun PA opportunities and coach and peer support Interactive internet based individual sessions with motivation and feedback messages CON: usual school activities	School	Self-efficacy (IPS) Enjoyment (IPS) Social support—teachers (ESS) Benefits of PA (IPS) Barriers to PA (IPS) Autonomous motivation (IPS)	Daily MVPA (min/h) Actigraph GT1M	T1: baseline T2: 6 months (post)	Health promotion model Self-determination theory
Santos et al., 2014 [85]	To assess the effectiveness of a peer-led healthy living program called ‘Healthy Buddies’ on weight gain and its determinants when disseminated at the provincial level to elementary school students	RCT Canada	9.3 (0.1) *n* = 647 Girls *n* = 330 (51%) Boys *n* = 317 Intervention *n* = 340 Control *n* = 307 - Younger *n* = 158 - Younger control *n* = 156 - Older *n* = 182 - Older control* n* = 151	For 8 months: 1 × /week 45-min lesson by researchers about healthy living to older children 1 × /week 30-min lesson by older children to younger children 2 × /weekly 30-min structured aerobic fitness sessions	School	Self-efficacy (IPS)	Daily (step count (steps/day) StepsCount Pedometer SC-01	T1: baseline T2: 8 months (post)	NA
Schneider et al., 2017 [86]	To evaluate a theory-based personalized exercise prescription to enhance motivation for being active and PA participation among adolescent reluctant exercisers	RCT USA	11.0 (0.4) *n* = 134 Girls *n* = 70 (52%) Boys *n* = 64 Reluctant, personalized intensity *n* = 34 Reluctant, moderate intensity *n* = 36 Latent personalized intensity *n* = 33 Latent moderate intensity *n* = 31 Analysed as: Reluctant personalized versus modified Latent personified versus modified	One session at self-selected feel-good intensity One session at set moderate intensity Both groups divided into latent and reluctant exercisers. Latent self-selected intensity and reluctant self-selected intensity used as intervention groups, with latent and reluctant moderate intensity as corresponding controls	School	Autonomous motivation (IPS)	Daily MVPA (min/day) Actigraph GT3X	T1: baseline T2: 8 weeks (post)	Dual-mode model of exercise-associated affect
Van Woudenberg et al., 2020 [87]	To investigate whether a social network intervention is more effective to promote PA, compared with a mass media intervention and no intervention	RCT Netherlands	11.3 (1.4) *n* = 446 Girls *n* = 241 (54%) Boys *n* = 205 MASS *n* = 48 SNI *n* = 63 Control *n* = 100	Vlogs produced and published by children in a study-specific app to known peers in the same school (familiar peers; Social network intervention) and to unknown peers in other schools (unfamiliar peers; mass media intervention)	School	Norms (INT) Autonomous motivation (IPS) Controlled motivation (IPS)	Daily (step count (steps/day) Fitbit tracker	T1: baseline T2: 1 week (post) T3: 5 weeks (follow-up)	Theory of planned behaviour; Self-determination theory
Vik et al., 2015 [88]	To evaluate whether the ‘UP4FUN’ intervention had an effect on reducing screen time and increasing breaking up sitting time (primary outcome), and also in changing specific personal determinants for these behaviours (e.g. awareness, attitude, self-efficacy regarding sedentary activities) and family environment determinants (e.g. parental practices, social environment, physical environment) [secondary outcome] among children and their parents in five countries across Europe	c-RCT Belgium Germany Greece Hungary Norway	Range = 10–12 11.2 (no SD) *n* = 3147 Girls *n* = 1605 (51%) Boys *n* = 1542 Intervention *n* = 1569 Control *n* = 1578	For 6 weeks, 2 × /week lessons 45 min at school by teachers trained by researchers Assignment to encourage child activity at school or home 1 × /week: newsletters to parents related to weekly theme	School	Self-efficacy (IPS) Attitudes (IPS) PA preference (IPS) Automaticity (IPS) PA knowledge (IPS)	Daily SB (h/day) Actigraph 7164	T1: baseline T2: 6 weeks (post)	Socio-ecological framework
Wang et al., 2017 [90]	To evaluate the effect of playing a health video game embedded with story immersion, Escape from Diab, on children’s diet and PA and to explore whether children immersed in Diab had greater positive outcomes	CT China	Range = 8–12 8–10 (52.5%) 11–12 (47.5%) *n* = 179 Girls *n* = 76 (43%) Boys *n* = 103 Intervention *n* = 95 Control *n* = 84	1 × /week for 9 weeks: > 40-min sessions playing a video game targeting knowledge about health behaviours eating and activity Goal setting at end of each weekly session	School	Self-efficacy (IPS) PA preference (IPS) Autonomous motivation (IPS) Controlled motivation (IPS)	Daily MVPA (min/day) Daily SB (min/day) Actigraph GT3X	T1: baseline T2: 8–10 weeks (post) T3: 16–20 weeks (follow-up)	Social cognitive theory; self-determination theory; elaboration-likelihood persuasion models
Zhang et al., 2020 [89]	To assess the effect of a facilitated behavioural intervention based on the extended theory of planned behaviour on psychological constructs and PA among adolescents over a period of eight weeks	RCT China	12.1 (0.3) *n* = 51 Girls *n* = 27 (53%) Boys *n* = 24 Intervention *n* = 24 Control *n* = 27	For 8 weeks, 1 × /week session 45 min, including five indoor courses and lectures and three outdoor basketball games	School Family/home	Self-efficacy (IPS) Attitudes (IPS) Norms (INT) PA outcome expectancies (IPS) Intentions (IPS) Perceived behavioural control (IPS)	Daily MVPA (%) Daily SB (%) Actigraph wGT3X-BT	T1: baseline T2: 8 weeks (post)	Theory of planned behaviour Social cognitive theory

*RCT* randomized controlled trial, *c-RCT* cluster-randomized controlled trial, *CT* controlled trial, *PA* physical activity, *SB* sedentary behaviour, *MVPA* moderate-vigorous physical activity, CON control group, *NA* not available, Determinant categories: *EBH* interpersonal behavioural, *IPS* individual psychological, *IPH* individual physiological, *ESS* interpersonal social support, *PEN* physical environmental, *IBH* individual behavioural, *INT* interpersonal

**Fig. 2:**
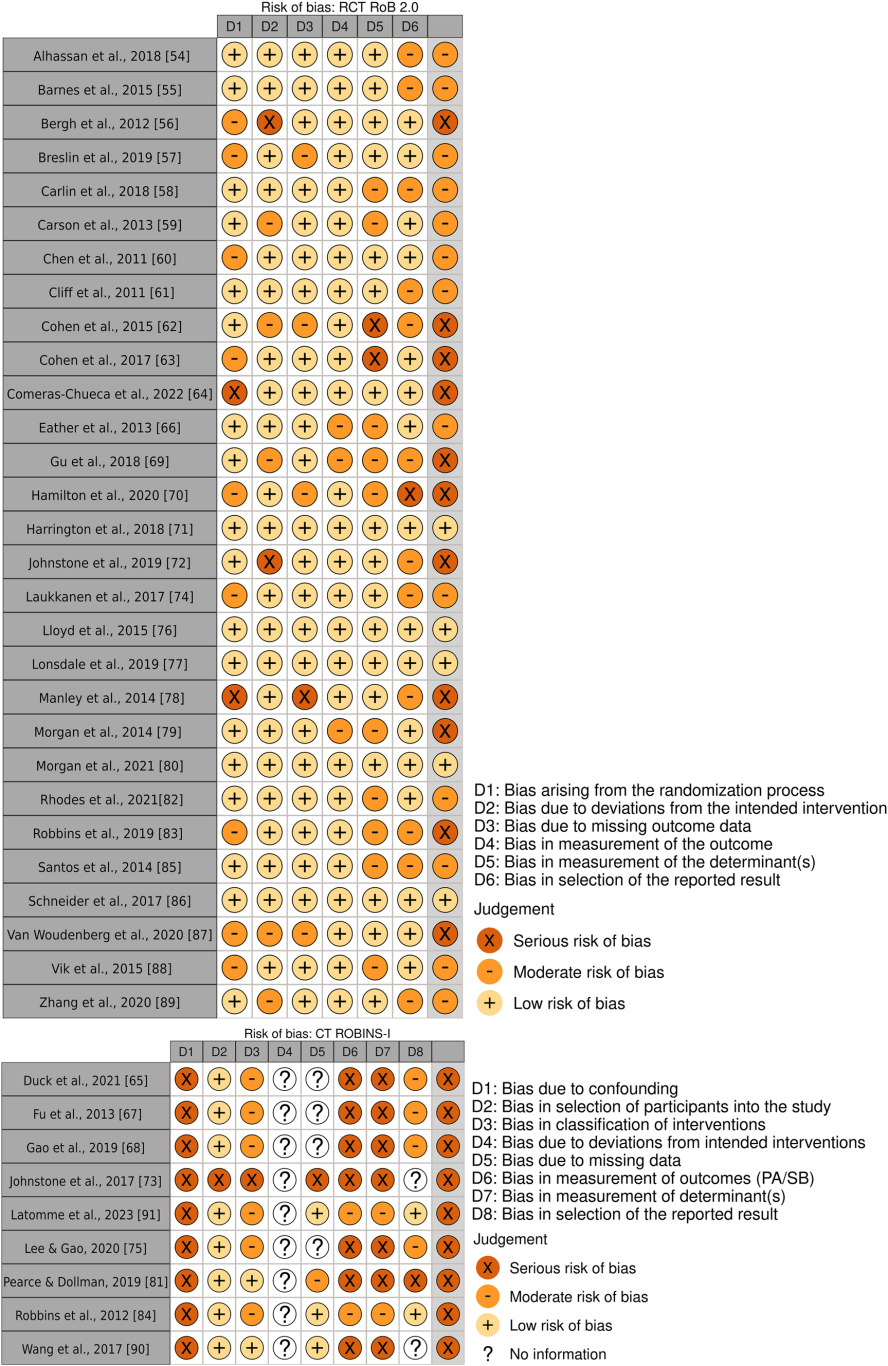
Results of risk of bias assessments

The risk of bias was overall high. Of the 29 RCTs, 11 were judged with a high risk of bias, 13 with a moderate risk of bias and 5 with a low risk of bias. Of the nine CTs, all were judged as having a serious risk of bias (Fig. 2). Based on GRADE, the overall quality of evidence was assessed as very low-to-moderate for PA/SB, and very low-to-high for the determinants. The GRADE assessments were incorporated into Tables 3, 4, 5, 6 and 7 and corresponding summaries of the GRADE assessments for the meta-analyses are provided in Supplementary file S2—Tables S1–S8.

**Table 3:** Results of the meta-analyses of the intervention effects on the determinants of PA/SB in the school setting in RCTs and CTs

	*n*	ES mean [95% CrI]	ES BF_10_	PB BF_10_	*τ* mean [95% CrI]	GRADE
School—post-RCT						
Amotivation	2	− 0.05 [− 0.40, 0.25]	0.10	0.56	0.11 [0.03, 0.35]	+ +
Attitudes	2	− 0.02 [− 0.36, 0.31]	0.09	0.56	0.12 [0.03, 0.38]	+ +
Autonomous motivation	6	− 0.14 [− 0.45, 0.16]	0.25	0.43	0.31 [0.16, 0.60]	+ +
Barriers to PA	2	0.04 [− 0.39, 0.39]	0.16	0.92	0.16 [0.04, 0.54]	+ +
Benefits of PA	2	0.12 [− 0.27, 0.46]	0.29	1.02	0.18 [0.04, 0.61]	+ +
Controlled motivation	3	0.04 [− 0.18, 0.22]	0.10	0.88	0.09 [0.03, 0.25]	+ +
Enjoyment	5	0.00 [− 0.22, 0.19]	0.78	1.47	0.12 [0.04, 0.29]	+ +
Motor competence	3	0.19 [− 0.48, 0.75]	0.41	1.12	0.43 [0.16, 1.08]	+
PA knowledge	2	0.16 [− 0.77, 1.37]	0.31	8.33	0.40 [0.04, 1.94]	+
PA outcome expectancies	2	0.27 [− 1.00, 1.35]	0.82	1.49	0.80 [0.10, 2.49]	+ +
Parenting for PA	2	− 0.03 [− 0.43, 0.33]	0.13	0.59	0.13 [0.03, 0.45]	+ +
Perception of physical environment	3	− 0.04 [− 0.86, 0.68]	0.42	1.79	0.37 [0.07, 1.02]	+ +
Self-efficacy	9	0.07 [− 0.19, 0.29]	0.14	0.44	0.29 [0.16, 0.50]	+ +
Social support—friends	5	− 0.04 [− 0.22, 0.10]	0.08	0.35	0.10 [0.03, 0.24]	+ +
Social support—parents	4	− 0.12 [− 0.33, 0.06]	0.26	0.55	0.13 [0.04, 0.34]	+
Social support—teachers	4	− 0.18 [− 0.43, 0.05]	0.62	0.45	0.23 [0.07, 0.55]	+
School—short-term RCT						
Social support—friends	2	− 0.25 [− 0.91, 0.38]	0.53	0.70	0.34 [0.08, 1.07]	+ +
School—post-CT						
Enjoyment	2	0.20 [− 0.57, 0.76]	0.48	1.70	0.25 [0.04, 0.95]	+ +
PA outcome expectancies	2	− 0.40 [− 0.91, 0.09]	1.57	0.62	0.28 [0.04, 1.01]	+ +
Self-efficacy	3	0.14 [− 0.31, 0.49]	0.31	0.99	0.19 [0.04, 0.57]	+ +
Social support—total	3	0.11 [− 0.60, 0.58]	0.36	0.21	0.20 [0.04, 0.62]	+ +

*n*  number of studies in meta-analysis, *ES* effect size, *95% CrI* 95% credible interval; PB BF_10_, Bayes factor (10) for publication bias; ES BF_10_, Bayes factor (10) for effect; *τ*, heterogeneity, *RCT* randomized controlled trial, *CT* controlled trial (non-randomized)

GRADE quality of evidence: + very low; +  + low; +  +  + moderate; +  +  +  + high

Timepoints: Immediately post-intervention, short-term maintenance (< 6 months post-intervention)

### Determinants

A total of 38 determinants were identified (Table 2), of which 30 were targeted in interventions conducted in the school setting, 15 in the family/home setting, 3 in the community setting, 15 in a combination of the school and family/home settings and 2 in a combination of the family/home and community settings. The determinants were categorized based on the social ecological model into individual psychological (*n* = 20), individual physiological (*n* = 4), individual behavioural (*n* = 1), interpersonal (*n* = 1), interpersonal behavioural (*n* = 4), interpersonal social support (*n* = 7) and physical environmental (*n* = 1). The most targeted determinants were self-efficacy (*n* = 17), enjoyment (*n* = 9), motor competence (*n* = 8), social support—parents (*n* = 8) and PA outcome expectancies (*n* = 7), autonomous motivation (*n* = 6), parenting for PA (*n* = 5), social support—teachers (*n* = 5) and social support—friends (*n* = 5).

For each setting, the results of the risk of bias and GRADE assessments were followed by the meta-analyses on the effects of the interventions on the determinants and on the PA/SB outcomes (Tables 3, 4, 5, 6 and 7). Determinants reported in single studies that were not pooled in meta-analyses were listed in the text. The complete numerical results for the pooled (RoBMA) and non-pooled determinants are presented in Supplementary file 1—Table 1, and PA and SB outcomes in Supplementary file 1—Table 2.

### School Setting

A total of 24 studies were conducted in the school setting, of which 16 were assessed as having a high risk of bias, 5 as some concern (moderate) and 3 as low risk of bias. The GRADE assessment indicated a very low-to-low quality of evidence for the determinants and a very low-to-high quality of evidence for the PA/SB outcomes (see Supplementary file 2—Tables S1–S3).

Seventeen RCTs were conducted in the school setting [56–59, 62, 63, 69–72, 77, 78, 83, 85–88]. For the RCTs, meta-analyses were conducted for 16 determinants, showing trivial-to-small effect sizes with trivial evidence for the presence of an effect for all. A meta-analysis was conducted for one determinant (social support—friends) for the short-term effect showing a negative small effect with trivial evidence for the presence of an effect (Table 3). Thirteen determinants were targeted in individual studies, including aerobic fitness [78], automaticity [88], intentions [71], moods and emotions [57], norms [87], PA preference [88], perceived athletic competence [63], physical self-perceptions [71], physical well-being [57], psychological well-being [57], psychosocial skills [70] and social support—total [83]. Seven CTs were conducted in the school setting [65, 67, 68, 73, 75, 84, 90]. For the CTs, meta-analyses were conducted for four determinants, showing trivial to small effect sizes with trivial evidence for the presence of and effect for all (Table 2). Eleven determinants were targeted in individual studies, including aerobic fitness [84], autonomous motivation [90], barriers to PA [84], benefits of PA [84], controlled motivation [90], exposure to PA models [84], motor competence [73], norms [84], PA preference [90], perceived athletic competence [67] and prosocial behaviour [65]. Forest plots can be found in Supplementary file 1—Figures S1–S3.

In the school setting, small effects were found for whole-day measures of PA in CTs and part-day measures of PA in RCTs at post-intervention, with trivial evidence for these effects. A moderate effect was found for part-day measures of SB in RCTs, with moderate evidence for the presence of the effect. The results for the PA and SB outcomes in the school setting are summarized in Table 4 and forest plots can be found in Supplementary file 1—Figures S4–S5.

**Table 4:** Results of the meta-analyses of the intervention effects on the PA and SB outcomes in the school setting in RCTs and CTs

	*n*	ES mean [95% CrI]	ES BF_10_	PB BF_10_	τ mean [95% CrI]	GRADE
Physical activity						
Whole-day—post-RCT	13	− 0.09 [− 0.37, 0.14]	0.15	1.11	0.24 [0.11, 0.45]	+
Part-day—post-RCT	4	0.29 [− 0.51, 0.97]	0.59	0.78	0.66 [0.30, 1.47]	+
Whole-day—short-term—RCT	2	− 0.18 [− 0.63, 0.25]	0.34	0.50	0.18 [0.04, 0.65]	+ + +
Part-day—short-term—RCT	1	0.10 [− 0.31, 0.52]	*	*	*	*
Whole-day—long-term—RCT	1	0.29 [− 0.31, 0.63]	*	*	*	*
Part-day—long-term—RCT	1	− 0.10 [− 0.29, 0.09]	*	*	*	*
Whole-day—post-CT	4	0.35 [− 0.38, 0.89]	0.82	0.74	0.53 [0.13, 1.25]	+
Part-day—post-CT	3	0.15 [− 1.20, 1.25]	0.60	0.95	1.03 [0.42, 2.5]	+ +
Sedentary behaviour						
Whole-day—post-RCT	4	0.05 [− 0.25, 0.37]	0.11	3.10	0.13 [0.03, 0.46]	+ + +
Part-day—post-RCT	3	0.58 [− 0.01, 0.91]	4.24	0.83	0.32 [0.05, 1.02]	+ + + +
Whole-day—short-term—RCT	1	0.42 [− 0.16, 0.99]	*	*	*	*
Part-day—long-term—RCT	1	0.67 [0.48, 0.87]	*	*	*	*
Part-day—short-term—RCT	1	0.00 [− 0.41, 0.42]	*	*	*	*
Whole-day—post-CT	1	0.53 [0.19, 0.86]	*	*	*	*
Part-day—post-CT	2	0.02 [− 1.78, 1.37]	0.71	1.21	1.29 [0.35, 3.76]	+ +

The PA and SB outcomes were measured over whole days (whole-day PA/SB) or parts of the days (part-day PA/SB). Post-intervention effects were measured immediately after intervention, short-term effects < 6 months after intervention, and long-term effects > 6 months after intervention

*n* = number of studies in meta-analysis, *ES* effect size, *95% CrI* 95% credible interval, *PB BF*_*10*_ Bayes factor (10) for publication bias, *ES BF*_*10*_ Bayes factor (10) for effect, *τ* heterogeneity, *RCT* randomized controlled trial, *CT* controlled trial (non-randomized)

GRADE Quality of evidence: + very low; +  + low; +  +  + moderate; +  +  +  + high

Timepoints: Immediately post-intervention, short-term maintenance (< 6 months post-intervention), long-term maintenance (> 6 months post-intervention)

### Family/Home Setting

Eight studies were conducted in the family/home setting, of which three were assessed as having a high risk of bias, three as moderate and two as low. The GRADE assessment indicated a low-to-high quality of evidence for the determinants and a low-to-moderate quality of evidence for the PA/SB outcomes (see Supplementary file 2—Tables S4–S6).

For the RCTs, meta-analyses were conducted for six determinants, showing trivial to medium effect sizes with trivial evidence for the presence of and effect for all, except parental PA modelling (ES = 0.69, ES BF_10_ = 3.49; Table 5). Nine determinants were targeted in individual studies, including attitudes [82], automaticity [82], intentions [82], motor competence [80], norms [82], PA knowledge [60], PA outcome expectancies [76], perceived athletic competence [80] and perceived behavioural control [82]. One CT was conducted in the family/home setting [91], reporting four determinants, including co-PA, family health climate, parental PA behaviour and parenting for PA. Seven RCTs were conducted in the family/home setting [55, 60, 74, 76, 79, 80, 82]. Forest plots can be found in Supplementary file 1—Figure S6.

**Table 5:** Results of the meta-analyses of the intervention effects on the determinants of PA/SB in the family/home setting in RCTs

	*n*	ES mean [95% CrI]	ES BF_10_	PB BF_10_	*τ* mean [95% CrI]	GRADE
Post-RCT						
Co-PA	3	0.37 [− 0.20, 0.76]	1.31	2.22	0.22 [0.04, 0.72]	+ + +
Parental PA modelling	2	0.69 [− 0.20, 1.19]	3.49	1.17	0.40 [0.04, 1.56]	+ + + +
Parental PA behaviour	2	0.27 [− 0.41, 0.81]	0.57	1.19	0.24 [0.04, 0.85]	+ +
Parenting for PA	3	0.02 [− 0.41, 0.39]	0.18	0.46	0.15 [0.04, 0.46]	+ + +
Self-efficacy	2	0.37 [− 0.51, 0.98]	0.90	2.93	0.26 [0.04, 0.99]	+ + +
Social support—parents	3	− 0.09 [− 0.64, 0.33]	0.21	0.45	0.15 [0.03, 0.46]	+ +

*n* number of studies in meta-analysis, *ES* effect size, *95% CrI* 95% credible interval, *PB BF*_*10*_ Bayes factor (10) for publication bias, *ES BF*_*10*_ Bayes factor (10) for effect, *τ* heterogeneity, *RCT* randomized controlled trial, *CT* controlled trial (non-randomized)

GRADE quality of evidence: + very low; +  + low; +  +  + moderate; +  +  +  + high

Timepoints: Immediately post-intervention

In the family/home setting, no presence of an effect was found for whole-day measures of PA and SB in the RCTs. The results for the PA and SB outcomes in the family/home setting are summarized in Table 6 and presented in forest plots in Supplementary file 1—Figure S7.

**Table 6:** Results of the meta-analyses of the intervention effects on the PA and SB outcomes in the family/home setting in RCTs and CTs

	*n*	ES mean [95% CrI]	ES BF_10_	PB BF_10_	*τ* mean [95% CrI]	GRADE
Physical activity						
Whole-day—post-RCT	7	0.22 [− 0.04, 0.43]	0.87	1.44	0.14 [0.03, 0.36]	+ + +
Whole-day—short-term—RCT	1	0.19 [− 0.37, 0.76]	*	*	*	*
Whole-day—long-term—RCT	1	− 0.43 [− 0.85, − 0.01]	*	*	*	*
Whole-day—post-CT	1	− 1.65 [− 2.2, − 1.11]	*	*	*	*
Sedentary behaviour						
Whole-day—post-RCT	2	0.02 [− 0.73, 0.59]	0.26	0.92	0.20 [0.04, 0.75]	+ +
Whole-day—long-term—RCT	1	0.00 [− 0.37, 0.37]	*	*	*	*
Whole-day—post-CT	1	3.17 [2.47, 3.87]	*	*	*	*

The PA and SB outcomes were measured over whole days (whole-day PA/SB) or parts of the days (part-day PA/SB). Post-intervention effects were measured immediately after intervention, short-term effects < 6 months after intervention, and long-term effects > 6 months after intervention

*n* number of studies in meta-analysis, *ES* effect size, *95% CrI* 95% credible interval, *PB BF*_*10*_ Bayes factor (10) for publication bias, *ES BF*_*10*_ Bayes factor (10) for effect, *τ* *BF*_*10*_ Bayes factor (10) for heterogeneity, *RCT* randomized controlled trial; *inestimable

GRADE quality of evidence: + very low; +  + low; +  +  + moderate; +  +  +  + high

Timepoints: Immediately post-intervention, short-term maintenance (< 6 months post-intervention), long-term maintenance (> 6 months post-intervention)

### Community Setting

One RCT was conducted in the community setting [64], which was assessed as having a high risk of bias. The study reported two determinants, of which only one was measured both pre- and post-intervention. A small effect of this intervention was found for motor competence and a large effect was found for the PA outcome.

### School and Family/Home Settings

Four studies were conducted in the combined school and family/home settings, of which one study was assessed as having a high risk of bias and three as some concerns (moderate). The GRADE assessment indicated a very low quality of evidence for self-efficacy and a high quality of evidence for whole-day PA (see Supplementary file 2—Tables S7–S8).

For the RCTs, one meta-analysis was conducted for self-efficacy, showing a negative trivial effect with trivial evidence for the presence of the effect and moderate evidence for the presence of heterogeneity (ES =  − 0.01 [− 0.92, 0.81]; ES BF_10_ = 0.32; *τ* = 0.55 [0.10, 1.53]; Supplementary file 1—Figure S8). Eleven determinants were targeted in individual studies, including attitudes [89], enjoyment [66], intentions [89], norms [89], PA outcome expectancies [89], PA preference [54], parental PA behaviour [54], perceived behavioural control [89], perception of physical environment [66], social support—friends [66] and social support—parents [66]. One CT was conducted in the school and family/home settings [81], reporting seven determinants, including barriers to PA, enjoyment, PA outcome expectancies, self-efficacy, self-management strategies, social support—parents and social support—teachers. Three RCTs were conducted in the combined school and family/home settings [54, 66, 89].

In the combined school and family/home settings, a small post-intervention effect was found for the whole-day measure of PA with trivial evidence for the effect in RCTs, the only outcome pooled. The results for the PA and SB outcomes in the combined school and family/home settings are summarized in Table 7 and the effect on PA is presented in a forest plot in Supplementary file 1—Figure S9.

**Table 7:** Results of the meta-analyses of the intervention effects on the PA and SB outcomes in the combined school and family/home settings in RCTs and CTs

	*n*	ES mean [95% CrI]	ES BF_10_	PB BF_10_	*τ* mean [95% CrI]	GRADE
Physical activity						
Whole-day—post-RCT	3	0.32 [− 0.27, 0.69]	0.96	4.04	0.21 [0.04, 0.66]	+ + + +
Whole-day—short-term—RCT	1	0.62 [0.35, 0.88]	*	*	*	*
Whole-day—post-CT	1	0.05 [− 0.44, 0.55]	*	*	*	*
Whole-day—short-term—CT	1	− 0.23 [− 0.72, 0.26]	*	*	*	*
Sedentary behaviour						
Whole-day—post-RCT	1	− 0.13 [− 0.68, 0.28]	*	*	*	*

The PA and SB outcomes were measured over whole days (whole-day PA/SB) or parts of the days (part-day PA/SB). Post-intervention effects were measured immediately after intervention, short-term effects < 6 months after intervention, and long-term effects > 6 months after intervention

*n* number of studies in meta-analysis, *ES* effect size, *95% CrI* 95% credible interval, *PB BF*_*10*_ Bayes factor (10) for publication bias, *ES BF*_*10*_ Bayes factor (10) for effect; *τ* BF_10_, Bayes factor (10) for heterogeneity; RCT, randomized controlled trial; *inestimable

GRADE quality of evidence: + very low; +  + low; +  +  + moderate; +  +  +  + high

Timepoints: Immediately post-intervention, short-term maintenance (< 6 months post-intervention)

### Community and Family/Home Settings

One RCT was conducted in the combined community and family/home settings [61], which was assessed as having a moderate risk of bias. Two determinants were reported, including motor competence and perceived athletic competence. A large post-intervention effect was found for motor competence and trivial effect was found for perceived athletic competence. Similarly, trivial post-intervention and long-term effects were found for PA.

## Discussion

The aims of the current systematic review and meta-analysis were to identify modifiable determinants of device-measured PA and SB targeted in available intervention studies with RCT and CT designs in children and early adolescents (5–12 years old) and quantify the effects of the interventions within their respective settings on the determinants of PA/SB, and the outcomes PA and SB. Overall, the results showed little effect of the interventions on PA, SB, and the modifiable determinants of PA/SB. The effects on the determinants were largely trivial-to-small with a few exceptions of moderate-to-large effects with moderate level of evidence for the observed effects, such as parental PA modelling in the family/home setting. The effects of the interventions on PA/SB were trivial-to-moderate, with the exception of moderate effect on the part-day measure of SB in school-based RCTs, with moderate level of evidence for the effect. Studies in the family/home setting showed relatively lower risk of bias compared to the other settings and a higher overall quality of evidence as indicated by GRADE. Although the interventions had little effects on the determinants of PA/SB and the PA and SB outcomes, the ones observed were found in RCTs but not in CTs, indicating that the higher level of methodological rigor associated with RCTs may have contributed to the outcomes.

In the school setting, the interventions showed mostly trivial effects on the determinants of PA and SB, and the PA and SB outcomes, except for the part-day measure of SB, which showed moderate evidence for the presence of an effect of ES = 0.58 and a moderate quality of evidence. The results in the school setting echoed previous reviews on the effects of school-based interventions on PA and SB, where a reduction in SB was observed but no increase in PA (MVPA, more specifically) [33, 92]. The school setting is particularly important for the promotion of healthy lifestyles as it is the setting in which children spend the most time outside of the family/home setting, in which they learn, socialize and have the opportunity to be physically active [93]. Several interventions in the school setting targeted the provision of PA opportunities and specific activities during school time (whether in class or during recess/breaks), or targeted teaching methods to include more PA elements in class (e.g., standing in class). An effect was found on part-day measured SB, but not any of the other PA/SB measures. The selection of whole-day measures of PA/SB in the current review was to enable comparison across studies, but it may be more important to consider outcome measures that correspond to the exposure within the interventions, such as a school-based intervention with a measure of PA/SB during school hours rather than a school-based intervention with a PA/SB measure that represents the whole day [10, 94].

In the family/home setting, the results showed moderate evidence for an effect of ES = 0.69 and a high quality of evidence for parental PA modelling. Only trivial evidence was found for the presence of any effects on PA and SB. The family/home setting showed consistently higher quality of evidence compared to the other settings. A previous meta-analysis on family-based interventions showed small significant effects of the interventions on PA [34]. A potential success of interventions conducted in the family/home setting alludes to an important role played by the immediate social environment, which largely involves parents and family members [26, 34]. Furthermore, it is plausible that interventions conducted in the family/home setting, particularly those including parents, could have a transferable effect to other settings [95]. For example, the determinants co-PA and parental PA modelling show promise and the recommended inclusion of parents in PA interventions in children echoes the results in previous literature [95]. The observed effects in the family/home setting in the current review are worth considering in future interventions. Finally, the community setting, and the combined settings showed no evidence for the presence of any effects on the determinants of PA or SB, likely due to a low number of included studies.

Regarding the theoretical underpinnings and contents of the interventions, a small number of theories were used as the bases for most of the interventions in the included studies. Social cognitive theory was the most cited theory (50% of included studies), of which self-efficacy and intention are central determinants of PA/SB [96]. Three studies did not mention a theoretical basis for their interventions, some of which were nevertheless still consistent with some of the well-established theories, targeting for example self-efficacy, perceived athletic competence, social support and PA outcome expectancies [67, 81, 85]. Four studies that did not mention a theoretical basis targeted motor competence and muscular/aerobic fitness, aiming to improve health outcomes along with increased PA/reduced SB [64, 72, 73, 97]. It has been argued that consistent small effects observed in the literature may reflect a failure of interventions to capture a wider array of determinants, and combinations of theories may lend a more comprehensive approach to interventions aiming to change PA/SB [8, 20]. It has been suggested that multilevel interventions based on the ecological approaches may provide a more comprehensive approach [26]. Ecological approaches, such as the social ecological model, emphasize the interaction between the individual and their social and physical environments and highlight that external factors can influence individual tendencies to engage in PA/SB, such as built environment, provision of safe outdoor areas, and provision/improvement of organized PA [26, 98, 99]. Three interventions in the included review were based on ecological theories, including ecological systems theory by Carson et al. [59] and socioecological model by Cohen et al. [62, 63]. As such, multilevel interventions based on ecological models, targeting determinants at individual and social and physical environment levels, warrant further exploration for future interventions. Emerging research in recent years has deviated from the largely social-cognitive approach with a focus on the individual in PA behaviour regulation (automaticity and habit) expanding on and challenging the current approach [100–103]. Three of the included studies [82, 86, 88] touched on such concepts and for a better understanding of them, further research is warranted.

The overall quality of evidence in the current review was assessed as low, with the exception of the family/home setting, which showed an especially high quality of evidence. The main components contributing to the low quality of evidence were the imprecision of studies, which denotes a wide variance among effect sizes (large credible intervals), i.e. low confidence in the results generated. Large credible intervals can be attributed to small sample sizes within studies and a small number of studies in a meta-analysis [104]. The small number of studies included in the meta-analyses may have been the main reason for the low confidence in the generated evidence.

In response to the quest of the European workplan on sport for evidence-based sport policies, the goal of the current review was to summarize and evaluate the available evidence on PA/SB and the modifiable determinants of PA/SB in children to inform a BESt to be used by policy makers and future researchers alike [9]. A strength of the current review was the use of the Bayesian approach in the meta-analyses, which provides a nuanced interpretation of the results, and particularly RoBMA, which yields publication bias-corrected effect sizes. Meta-analyses based on the frequentist statistical approach were also conducted for the current review and have been reported in the Supplementary file 1—Table S1. It is important to note that the results of RoBMA and the frequentist meta-analyses are not comparable due to the difference in inference methods and the publication bias adjustment in RoBMA. Another strength of the current review was the attention to the quality of the produced evidence, which is key for the interpretation of the results and translation of the evidence to policy (e.g. BESt). A limitation in the current review was related to the exclusive focus on modifiable determinants limited the breadth of the current review, as additional studies may have been included that target non-modifiable determinants. However, since the current review had the goal to provide evidence for future interventions and policy, determinants were sought that were modifiable and thus possible to manipulate in interventions. Furthermore, studies including clinical populations were excluded from the current review. Among the included studies, there were studies including participants with overweight and/or obesity (the whole sample or parts of the sample) that did not explicitly disclose any medical conditions. However, it could be that participants with overweight/obesity may have unknown medical conditions that affect their ability to engage in PA. Additionally, participants with overweight/obesity may be more motivated to take part in PA/SB interventions and, as a result, may respond particularly well to the intervention [105]. Such participant characteristics must be taken into account when interpreting the results of the meta-analyses. Moreover, the current review targeted RCTs and CTs as having robust designs that produce a high level of evidence and can provide indication of causality due to repeated measures of the determinants and the PA and SB outcomes. However, due to the controlled nature of RCTs and CTs, their application in the real world may be difficult and their chosen methods may not be ecologically valid. Therefore, the application of PA and SB interventions would require accurate translation to policy, adaptation of methods to target environments and theory-informed methods for implementation in their respective settings [106]. Finally, the studies with both PA and SB as outcomes did not distinguish between determinants of PA or SB, but rather aimed to manipulate their targeted determinants and measured PA and SB simultaneously. As such, it was not possible to make any distinctions between determinants of PA and SB in the current review.

## Conclusions

Taken together, the results of the current systematic review and meta-analysis suggest that the included interventions have not been effective in changing PA/SB or the determinants of PA/SB overall and the few changes shown were not sustained over time. Parental PA modelling in the family/home setting indicated a moderate effect and a moderate level of evidence, which may suggest that parental involvement may be key to increasing PA/reducing SB in children. The part-day measure of SB in the school setting also indicated a moderate effect and a moderate level of evidence, which may suggest that an outcome measure (school-based measure) that corresponds to the exposure (school-based intervention) may be an important consideration. Despite the lack of effects overall, it seems that the family/home setting shows promise with consistently higher quality of evidence compared with the school and community settings and the combinations of settings, which is an important finding. The current review also helps uncover the breadth of methodological variability and aspects of PA research in children which need to be addressed in future research, relating to the selection of both determinants and behaviour change techniques. The results of the current review shed light on the continued need for refined theory-based interventions and the continued need to deepen our understanding of how determinants underpin PA/SB and provide a relevant basis for evidence-based policies and interventions. Gaps were identified that relate to determinants on the physical environmental level, the integration of determinants based on an ecological approach and the emergence of research on automaticity and habit of PA behaviour on the individual level that warrant further investigation.

## Supplementary Information

Below is the link to the electronic supplementary material.

Supplementary file 1: Complete results, including search strategy, description of data synthesis methods for frequentist analysis, complete results for pooled and un-pooled effects on determinants of PA/SB and PA/SB, and forest plots for meta-analyses

Supplementary file 2: GRADE assessments

## Declarations

### Availability of Data and Materials

Not applicable. No primary data were generated for the current review.
