# Supplementary material for: DE-PASS Best Evidence Statement (BESt): A Systematic Review and Meta-analysis on the Effectiveness of Trials on Device-Measured Physical Activity and Sedentary Behaviour and Their Determinants in Children Aged 5–12 Years
Source: Sports Med. 2024 Dec 6;55(2):419–58. doi: 10.1007/s40279-024-02136-8 (PMC11947004; doi:10.1007/s40279-024-02136-8)
Supplement: Supplementary file 1 — Complete results, including search strategy, description of data synthesis methods for frequentist analysis, complete results for pooled and un-pooled effects on determinants of PA/SB and PA/SB, and forest plots for meta-analyses [file 40279_2024_2136_MOESM1_ESM.docx]

**Data synthesis – Frequentist meta-analysis**

Using the classing frequentist statistical approach, meta-analyses were conducted with random effects models, as heterogeneity was expected among the included studies due to the nature of PA/SB interventions (Von Hippel, 2015). The data for change from pre- to post-intervention were used to calculate the between-group difference (intervention and control). For the interpretation of effect sizes, the following benchmarks were used: <0.2 trivial effect, 0.2 < 0.5 small effect, 0.5 < 0.8 medium effect, and *d* ≥ 0.80 large effect (Cohen, 1988). Heterogeneity was identified using I^2^ and the following benchmarks were used: I^2^ < 25% low heterogeneity, 25% < I^2^ < 50% moderate heterogeneity and I^2^ > 75% high heterogeneity (Borenstein et al., 2017; Higgins et al., 2003). Publication bias was estimated using Egger’s test (Sterne et al., 2011) for meta-analyses including 10 or more studies as publication bias can be substantially biased in meta-analyses with small (<10) numbers of studies (Page et al., 2022, p. 13; Sterne et al., 2011). The level for statistical significance was set to α<0.05. The results of the frequentist approach were reported in Supplementary tables 2-3.

Borenstein, M., Higgins, J. P. T., Hedges, L. V., & Rothstein, H. R. (2017). Basics of meta-analysis: I2 is not an absolute measure of heterogeneity. *Research Synthesis Methods*, *8*(1), 5–18. https://doi.org/10.1002/jrsm.1230

Cohen, J. (1988). *Statistical power analysis for the behavioral sciences*. Lawrence Erlbaum Associates Inc.

Higgins, J., Thompson, S. G., Deeks, J. J., & Altman, D. G. (2003). Measuring inconsistency in meta-analyses. *British Medical Journal*, *327*, 557–560. https://doi.org/10.1136/bmj.327.7414.557

Page, M., Higgins, J., & Sterne, J. (2022). Chapter 13: Assessing risk of bias due to missing results in a synthesis. In J. Higgins, J. Thomas, J. Chandler, M. Cumpston, T. Li, & M. Page (Eds.), *Cochrane Handbook for Systematic Reviews of Interventions* (6.3).

Sterne, J. A. C., Sutton, A. J., Ioannidis, J. P. A., Terrin, N., Jones, D. R., Lau, J., Carpenter, J., Rucker, G., Harbord, R. M., Schmid, C. H., Tetzlaff, J., Deeks, J. J., Peters, J., Macaskill, P., Schwarzer, G., Duval, S., Altman, D. G., Moher, D., & Higgins, J. P. T. (2011). Recommendations for examining and interpreting funnel plot asymmetry in meta-analyses of randomised controlled trials. *BMJ*, *343*(jul22 1), d4002–d4002. https://doi.org/10.1136/bmj.d4002

Von Hippel, P. T. (2015). The heterogeneity statistic I2 can be biased in small meta-analyses. *BMC Medical Research Methodology*, *15*(1), 35. https://doi.org/10.1186/s12874-015-0024-z

Table S1. Complete results for pooled and un-pooled effects on determinants of PA/SB in RCTs and CTs, respectively. Results for both meta-analyses based on the classic frequentist and Bayesian (Robust Bayesian meta-analysis; RoBMA) approaches Effects of the Bayesian approach were adjusted for the reported publication bias. Includes post-intervention effects and maintenance effects at short-term (<6 months post-intervention measure) and long-term (>6 months post-intervention measure).

|  |  | **Frequentist** | | |  | **Bayesian** | | | |
| --- | --- | --- | --- | --- | --- | --- | --- | --- | --- |
|  | **n** | **SMD**  **[95%CI]** | **I^2^**  **(%)** | **p** |  | **ES**  **[95%CI]** | **ES BF_10_** | **PB BF_10_** | **τ mean [95%CI]** |
| **School** | |  |  |  |  |  |  |  |  |
| **Post - CT** | |  |  |  |  |  |  |  |  |
| Aerobic fitness | 1 | 0.31 [-0.18, 0.81] | * | * |  | * | * | * | * |
| Autonomous motivation | 1 | 0.14 [-0.17, 0.44] | * | * |  | * | * | * | * |
| Barriers to PA | 1 | -0.20 [-0.68, 0.28] | * | * |  | * | * | * | * |
| Benefits of PA | 1 | -0.22 [-0.7, 0.26] | * | * |  | * | * | * | * |
| Controlled motivation | 1 | 0.04 [-0.27, 0.34] | * | * |  | * | * | * | * |
| Enjoyment | 2 | 0.35 [-0.09, 0.79] | 55.13 | 0.120 |  | 0.2 [-0.57, 0.76] | 0.48 | 1.7 | 0.25 [0.04, 0.95] |
| Exposure to PA models | 1 | -0.32 [-0.82, 0.18] | * | * |  | * | * | * | * |
| Motor competence | 1 | 0.58 [0.11, 1.06] | * | * |  | * | * | * | * |
| Norms | 1 | -0.36 [-0.86, 0.13] | * | * |  | * | * | * | * |
| PA outcome expectancies | 2 | -0.38 [-0.69, -0.07] | 27.06 | **0.016** |  | -0.4 [-0.91, 0.09] | 1.57 | 0.62 | 0.28 [0.04, 1.01] |
| PA preference | 1 | 0.27 [-0.03, 0.58] | * | * |  | * | * | * | * |
| Perceived athletic competence | 1 | 0.26 [-0.24, 0.77] | * | * |  | * | * | * | * |
| Prosocial behaviour | 1 | 0.34 [-0.33, 1.01] | * | * |  | * | * | * | * |
| Self-efficacy | 3 | 0.20 [-0.05, 0.44] | 31.85 | 0.114 |  | 0.14 [-0.31, 0.49] | 0.31 | 0.99 | 0.19 [0.04, 0.57] |
| Social support - total | 3 | 0.27 [-0.07, 0.62] | 52.75 | 0.119 |  | 0.11 [-0.6, 0.58] | 0.36 | 0.21 | 0.20 [0.04, 0.62] |
| **Post - RCT** | |  |  |  |  | * |  |  |  |
| Aerobic fitness | 1 | 0.27 [-0.12, 0.66] | * | * |  | * | * | * | * |
| Amotivation | 2 | -0.03 [-0.1, 0.05] | 0.00 | 0.518 |  | -0.05 [-0.4, 0.25] | 0.1 | 0.56 | 0.11 [0.03, 0.35] |
| Attitudes | 2 | 0.00 [-0.11, 0.11] | 65.68 | 0.950 |  | -0.02 [-0.36, 0.31] | 0.09 | -0.56 | 0.12 [0.03, 0.38] |
| Automaticity | 1 | -0.01 [-0.08, 0.06] | * | * |  | * | * | * | * |
| Autonomous motivation | 6 | -0.12 [-0.37, 0.14] | 93.28 | 0.365 |  | -0.14 [-0.45, 0.16] | 0.25 | 0.43 | 0.31 [0.16, 0.60] |
| Barriers to PA | 2 | 0.09 [-0.07, 0.24] | 31.4 | 0.270 |  | 0.04 [-0.39, 0.39] | 0.16 | 0.92 | 0.16 [0.04, 0.54] |
| Benefits of PA | 2 | 0.16 [0.06, 0.25] | 0.00 | **0.001** |  | 0.12 [-0.27, 0.46] | 0.29 | 1.02 | 0.18 [0.04, 0.61] |
| Controlled motivation | 3 | 0.06 [-0.01, 0.13] | 0.00 | 0.109 |  | 0.05 [-0.18, 0.22] | 0.1 | 0.88 | 0.09 [0.03, 0.25] |
| Enjoyment | 5 | 0.04 [-0.10, 0.18] | 74.99 | 0.621 |  | 0 [-0.22, 0.19] | 0.78 | 1.47 | 0.12 [0.04, 0.29] |
| Intentions | 1 | -0.04 [-0.15, 0.07] | * | * |  | * | * | * | * |
| Moods & emotions | 1 | -0.21 [-0.37, -0.05] | * | * |  | * | * | * | * |
| Motor competence | 3 | 0.27 [-0.19, 0.73] | 90.15 | 0.255 |  | 0.19 [-0.48, 0.75] | 0.41 | 1.12 | 0.43 [0.16, 1.08] |
| Norms | 1 | 0.03 [-0.16, 0.21] | * | * |  | * | * | * | * |
| PA knowledge | 2 | 0.97 [-0.11, 3.00] | 96.32 | 0.347 |  | 0.17 [-0.77, 1.37] | 0.31 | **8.33** | 0.40 [0.04, 1.94] |
| PA outcome expectancies | 2 | 0.69 [-0.67, 2.04] | 98.66 | 0.320 |  | 0.27 [-1, 1.35] | 0.82 | 1.49 | 0.80 [0.10, 2.49] |
| PA preference | 1 | -0.03 [-0.10, 0.04] | * | * |  | * | * | * | * |
| Parenting for PA | 2 | -0.01 [-0.15, 0.13] | 0.00 | 0.871 |  | -0.03 [-0.43, 0.33] | 0.13 | 0.59 | 0.13 [0.03, 0.45] |
| Perception of physical environment | 3 | 0.23 [-0.30, 0.77] | 96.59 | 0.388 |  | -0.04 [-0.86, 0.68] | 0.42 | 1.79 | 0.37 [0.07, 1.02] |
| Perception of physical environment | 1 | -0.12 [-0.23, -0.01] | * | * |  | * | * | * | * |
| Physical self-perceptions | 1 | 0.02 [-0.09, 0.13] | * | * |  | * | * | * | * |
| Physical well-being | 1 | 0.04 [-0.12, 0.20] | * | * |  | * | * | * | * |
| Psychological well-being | 1 | -0.08 [-0.24, 0.08] | * | * |  | * | * | * | * |
| Psychosocial skills | 1 | 0.82 [0.17, 1.48] | * | * |  | * | * | * | * |
| Self-efficacy | 9 | 0.09 [-0.12, 0.30] | 93.59 | 0.408 |  | 0.07 [-0.19, 0.29] | 0.14 | 0.44 | 0.29 [0.16, 0.50] |
| Social support - Friends | 5 | -0.03 [-0.13, 0.07] | 42.68 | 0.583 |  | -0.04 [-0.22, 0.10] | 0.08 | 0.35 | 0.10 [0.03, 0.24] |
| Social support - Parents | 4 | -0.11 [-0.19, -0.02] | 0.01 | **0.011** |  | -0.12 [-0.33, 0.06] | 0.26 | 0.55 | 0.13 [0.04, 0.34] |
| Social support - Teachers | 4 | -0.17 [-0.36, 0.01] | 83.67 | 0.066 |  | -0.18 [-0.43, 0.05] | 0.62 | 0.45 | 0.23 [0.07, 0.55] |
| Social support - total | 1 | 0.28 [0.18, 0.38] | * | * |  | * | * | * | * |
| **Short-term - CT** | |  |  |  |  |  |  |  |  |
| Autonomous motivation | 1 | 0.12 [-0.18, 0.43] | * | * |  | * | * | * | * |
| Controlled motivation | 1 | -0.02 [-0.32, 0.29] | * | * |  | * | * | * | * |
| PA preference | 1 | 0.13 [-0.18, 0.43] | * | * |  | * | * | * | * |
| Self-efficacy | 1 | 0.28 [-0.03, 0.58] | * | * |  | * | * | * | * |
| **Short-term - RCT** |  |  |  |  |  | * |  |  |  |
| Autonomous motivation | 1 | 0.11 [-0.07, 0.30] | * | * |  | * | * | * | * |
| Barriers to PA | 1 | 0.09 [-0.20, 0.38] | * | * |  | * | * | * | * |
| Benefits of PA | 1 | 0.09 [-0.20, 0.38] | * | * |  | * | * | * | * |
| Controlled motivation | 1 | -0.09 [-0.28, 0.09] | * | * |  | * | * | * | * |
| Moods & emotions | 1 | -0.23 [-0.39, -0.06] | * | * |  | * | * | * | * |
| Norms | 1 | -0.08 [-0.26, 0.11] | * | * |  | * | * | * | * |
| Parenting for PA | 1 | -0.08 [-0.25, 0.08] | * | * |  | * | * | * | * |
| Physical well-being | 1 | 0.08 [-0.09, 0.24] | * | * |  | * | * | * | * |
| Psychological well-being | 1 | -0.05 [-0.28, 0.18] | * | * |  | * | * | * | * |
| Self-efficacy | 1 | -0.19 [-0.48, 0.10] | * | * |  | * | * | * | * |
| Social support - Friends | 2 | -0.18 [-0.65, 0.29] | 87.52 | 0.454 |  | -0.25 [-0.91, 0.38] | 0.53 | 0.70 | 0.34 [0.08, 1.07] |
| Social support - Parents | 1 | 0.13 [-0.16, 0.42] | * | * |  | * | * | * | * |
| **Long-term - RCT** |  |  |  |  |  |  |  |  |  |
| Amotivation | 1 | -0.04 [-0.16, 0.09] | * | * |  | * | * | * | * |
| Autonomous motivation | 1 | -0.57 [-0.69, -0.44] | * | * |  | * | * | * | * |
| Controlled motivation | 1 | 0.09 [-0.04, 0.21] | * | * |  | * | * | * | * |
| Motor competence | 1 | 0.51 [0.30, 0.73] | * | * |  | * | * | * | * |
| Social support - Teachers | 1 | -0.17 [-0.29, -0.05] | * | * |  | * | * | * | * |
| **Family/Home** | |  |  |  |  |  |  |  |  |
| **Post - RCT** | |  |  |  |  |  |  |  |  |
| Attitudes | 1 | -0.08 [-0.52, 0.36] | * | * |  | * | * | * | * |
| Automaticity | 1 | -0.14 [-0.58, 0.30] | * | * |  | * | * | * | * |
| Co-PA | 3 | 0.48 [0.24, 0.71] | 0.00 | **<.001** |  | 0.37 [-0.20, 0.76] | 1.31 | 2.22 | 0.22 [0.04, 0.72] |
| Intentions | 1 | 0.10 [-0.21, 0.41] | * | * |  | * | * | * | * |
| Motor competence | 1 | 1.35 [1.04, 1.67] | * | * |  | * | * | * | * |
| Norms | 1 | -0.04 [-0.48, 0.40] | * | * |  | * | * | * | * |
| PA knowledge | 1 | 0.61 [0.05, 1.18] | * | * |  | * | * | * | * |
| PA outcome expectancies | 1 | 0.33 [-0.26, 0.92] | * | * |  | * | * | * | * |
| Parental modelling | 2 | 0.84 [0.44, 1.24] | 36.31 | **<.001** |  | 0.69 [-0.20, 1.19] | **3.49** | 1.12 | 0.40 [0.04, 1.56] |
| Parental PA behaviour | 2 | 0.38 [0.02, 0.74] | 0.00 | **0.036** |  | 0.27 [-0.41, 0.81] | 0.57 | 1.19 | 0.24 [0.04, 0.85] |
| Parenting for PA | 3 | 0.07 [-0.18, 0.32] | 0.00 | 0.593 |  | 0.02 [-0.41, 0.39] | 0.18 | 0.46 | 0.15 [0.04, 0.46] |
| Perceived athletic competence | 1 | 0.14 [-0.15, 0.44] | * | * |  | * | * | * | * |
| Perceived behavioural control | 1 | -0.07 [-0.51, 0.37] | * | * |  | * | * | * | * |
| Self-efficacy | 2 | 0.59 [0.18, 1.00] | 0.00 | **0.005** |  | 0.37 [-0.51, 0.98] | 0.90 | 2.93 | 0.26 [0.04, 0.99] |
| Social support - Parents | 3 | -0.04 [-0.34, 0.25] | 0.00 | 0.784 |  | -0.09 [-0.64, 0.33] | 0.21 | 0.45 | 0.15 [0.03, 0.46] |
| **Short-term - RCT** | |  |  |  |  |  |  |  |  |
| Parental PA behaviour | 1 | 0.22 [-0.40, 0.84] | * | * |  | * | * | * | * |
| Parenting for PA | 1 | -0.05 [-0.67, 0.57] | * | * |  | * | * | * | * |
| Social support - Parents | 1 | -0.06 [-0.68, 0.56] | * | * |  | * | * | * | * |
| Family/Home - Long-term - RCT |  |  |  |  |  |  |  |  |  |
| Social support - Parents | 1 | 0.04 [-0.37, 0.45] | * | * |  | * | * | * | * |
| **Post – CT** | |  |  |  |  |  |  |  |  |
| Co-PA | 1 | 5.20 [4.23, 6.16] | * | * |  | * | * | * | * |
| Family Health Climate | 1 | 0.00 [-0.47, 0.47] | * | * |  | * | * | * | * |
| Parental PA behaviour | 1 | 2.43 [1.82, 3.05] | * | * |  | * | * | * | * |
| Parenting for PA | 1 | 1.60 [1.06, 2.14] | * | * |  | * | * | * | * |
| **Community** | |  |  |  |  |  |  |  |  |
| **Post – RCT** |  |  |  |  |  |  |  |  |  |
| Muscle strength | 1 | 0.18 [-0.63, 1.00] | * | * |  | * | * | * | * |
| **Post – CT** | |  |  |  |  |  |  |  |  |
| Motor competence | 1 | 0.34 [-0.61, 1.29] | * | * |  | * | * | * | * |
| Aerobic fitness | 1 | 0.53 [-0.23, 1.29] | * | * |  | * | * | * | * |
| **School & Family/Home** | |  |  |  |  |  |  |  |  |
| **Post - CT** | |  |  |  |  |  |  |  |  |
| Barriers to PA | 1 | 0.43 [0.06, 0.80] | * | * |  | * | * | * | * |
| Enjoyment | 1 | 0.13 [-0.24, 0.50] | * | * |  | * | * | * | * |
| PA outcome expectancies | 1 | -0.45 [-0.83, -0.08] | * | * |  | * | * | * | * |
| Self-efficacy | 1 | -0.11 [-0.48, 0.26] | * | * |  | * | * | * | * |
| Self-management strategies | 1 | 0.12 [-0.25, 0.49] | * | * |  | * | * | * | * |
| Social support - Parents | 1 | 0.09 [-0.28, 0.46] | * | * |  | * | * | * | * |
| Social support - Teachers | 1 | 0.27 [-0.10, 0.64] | * | * |  | * | * | * | * |
| **Post - RCT** | |  |  |  |  |  |  |  |  |
| Attitudes | 1 | 0.17 [-0.38, 0.72] | * | * |  | * | * | * | * |
| Enjoyment | 1 | -0.28 [-0.54, -0.02] | * | * |  | * | * | * | * |
| Intentions | 1 | 0.87 [0.30, 1.45] | * | * |  | * | * | * | * |
| Norms | 1 | 0.43 [-0.13, 0.98] | * | * |  | * | * | * | * |
| PA outcome expectancies | 1 | 0.52 [-0.04, 1.08] | * | * |  | * | * | * | * |
| PA preference | 1 | -0.07 [-0.56, 0.42] | * | * |  | * | * | * | * |
| Parental PA behaviour | 1 | 0.15 [-0.34, 0.64] | * | * |  | * | * | * | * |
| Perceived behavioural control | 1 | 0.44 [-0.12, 1.00] | * | * |  | * | * | * | * |
| Perception of physical environment | 1 | -0.78 [-1.05, -0.51] | * | * |  | * | * | * | * |
| Self-efficacy | 3 | 0.09 [-0.80, 0.97] | 91.86 | 0.850 |  | -0.01 [-0.92, 0.81] | 0.32 | 0.86 | 0.55 [0.10, 1.53] |
| Social support - Friends | 1 | -0.08 [-0.35, 0.18] | * | * |  | * | * | * | * |
| Social support - Parents | 1 | -0.03 [-0.29, 0.23] | * | * |  | * | * | * | * |
| **Short-term - CT** | |  |  |  |  |  |  |  |  |
| Barriers to PA | 1 | 0.26 [-0.15, 0.68] | * | * |  | * | * | * | * |
| Enjoyment | 1 | 0.22 [-0.19, 0.63] | * | * |  | * | * | * | * |
| PA outcome expectancies | 1 | -0.25 [-0.67, 0.16] | * | * |  | * | * | * | * |
| Self-efficacy | 1 | 0.00 [-0.41, 0.41] | * | * |  | * | * | * | * |
| Self-management strategies | 1 | 0.44 [0.02, 0.85] | * | * |  | * | * | * | * |
| Social support - Parents | 1 | 0.18 [-0.23, 0.59] | * | * |  | * | * | * | * |
| Social support - Teachers | 1 | 0.62 [0.20, 1.04] | * | * |  | * | * | * | * |
| **Short-term - RCT** | |  |  |  |  | * |  |  |  |
| Enjoyment | 1 | 0.26 [0.00, 0.52] | * | * |  | * | * | * | * |
| Perception of physical environment | 1 | -1.12 [-1.4, -0.84] | * | * |  | * | * | * | * |
| Self-efficacy | 1 | 0.04 [-0.22, 0.30] | * | * |  | * | * | * | * |
| Social support - Friends | 1 | -0.10 [-0.37, 0.16] | * | * |  | * | * | * | * |
| Social support - Parents | 1 | -0.17 [-0.43, 0.09] | * | * |  | * | * | * | * |
| **Community & Family/Home** | |  |  |  |  |  |  |  |  |
| **Post - RCT** | |  |  |  |  |  |  |  |  |
| Motor competence | 1 | 0.88 [0.38, 1.38] | * | * |  | * | * | * | * |
| Perceived athletic competence | 1 | 0.07 [-0.4, 0.53] | * | * |  | * | * | * | * |
| **Long-term - RCT** |  |  |  |  |  |  |  |  |  |
| Motor competence | 1 | 0.20 [-0.28, 0.68] | * | * |  | * | * | * | * |
| Perceived athletic competence | 1 | 0.12 [-0.34, 0.59] | * | * |  | * | * | * | * |
| n=number of studies in meta-analysis; SMD=Standardized mean difference; CI95%=95% confidence interval; I2=Heterogeneity; ES=Effect size; CrI95%=Credible interval; PB BF_10_=Bayes factor (10) for publication bias; ES BF_10_=Bayes factor (10) for effect; τ BF_10_=Bayes factor (10) for heterogeneity; RCT=Randomized controlled trial; CT=(non-randomized) Controlled trial | | | | | | | | | |
| P-value in bold digits indicate statistically significant effects. Bayes factors in bold digits indicate moderate evidence or higher. | | | | | | | | | |

Table S2. Complete results for pooled and un-pooled effects on PA and SB in RCTs and CTs, respectively. Results for both meta-analyses based on the classic frequentist and Bayesian (Robust Bayesian meta-analysis; RoBMA) approaches. Effects of the Bayesian approach were adjusted for the reported publication bias. Includes post-intervention effects and maintenance effects at short-term (<6 months post-intervention measure) and long-term (>6 months post-intervention measure).

|  |  | **Frequentist** | | |  | **Bayesian** | | | |  |
| --- | --- | --- | --- | --- | --- | --- | --- | --- | --- | --- |
|  | **n** | **SMD [95%CI]** | **I2 (%)** | **p** |  | **ES [95%CI]** | **ES BF10** | **PB BF10** | **τ [95%CI]** |  |
| **School** |  |  |  |  |  |  |  |  |  |  |
| **Physical activity** |  |  |  |  |  |  |  |  |  |  |
| Whole-day - Post - CT | 4 | 0.44 [-0.14, 1.03] | 71.21 | 0.139 |  | 0.35 [-0.38, 0.89] | 0.82 | 0.74 | 0.53 [0.13, 1.25] |  |
| Whole-day - Post - RCT | 13 | 0.00 [-0.16, 0.17] | 85.74 | 0.972 |  | -0.09 [-0.37, 0.14] | 0.15 | 1.11 | 0.24 [0.11, 0.45] |  |
| Part-day - Post - CT | 3 | 0.48 [-0.94, 1.90] | 96.42 | 0.507 |  | 0.15 [-1.20, 1.25] | 0.60 | 0.95 | 1.03 [0.42, 2.50] |  |
| Part-day - Post - RCT | 4 | 0.41 [-0.24, 1.07] | 94.77 | 0.217 |  | 0.29 [-0.51, 0.97] | 0.59 | 0.78 | 0.66 [0.30, 1.47] |  |
| Whole-day - Short-term - RCT | 2 | -0.16 [-0.34, 0.02] | 0.00 | 0.076 |  | -0.18 [-0.63, 0.25] | 0.34 | 0.5 | 0.18 [0.04, 0.65] |  |
| Part-day – Short-term – RCT | 1 | 0.10 [-0.31, 0.52] | * | * |  | * | * | * | * |  |
| Whole-day – Long-term – RCT | 1 | 0.29 [-0.31, 0.63] | * | * |  | * | * | * | * |  |
| Part-day – Long-term – RCT | 1 | -0.10 [-0.29, 0.09] | * | * |  | * | * | * | * |  |
| **Sedentary behaviour** |  |  |  |  |  |  |  |  |  |  |
| Whole-day – Post – CT | 1 | 0.53 [0.19, 0.86] | * | * |  | * | * | * | * |  |
| Whole-day - Post - RCT | 4 | 0.06 [0.00, 0.12] | 0.00 | 0.033 |  | 0.05 [-0.25, 0.37] | 0.11 | 3.10 | 0.13 [0.03, 0.46] |  |
| Part-day - Post - CT | 2 | 0.49 [-1.89, 2.87] | 98.02 | 0.686 |  | 0.02 [-1.78, 1.37] | 0.71 | 1.21 | 1.29 [0.35, 3.76] |  |
| Part-day - Post - RCT | 3 | 0.63 [0.35, 0.92] | 63.43 | <.001 |  | 0.58 [-0.01, 0.91] | 4.24 | 0.83 | 0.32 [0.05, 1.02] |  |
| Whole-day – Short-term – RCT | 1 | 0.42 [-0.16, 0.99] | * | * |  | * | * | * | * |  |
| Part-day – Long-term – RCT | 1 | 0.67 [0.48, 0.87] | * | * |  | * | * | * | * |  |
| Part-day – Short-term – RCT | 1 | 0.00 [-0.41, 0.42] | * | * |  | * | * | * | * |  |
| **Family Home** |  |  |  |  |  |  |  |  |  |  |
| **Physical activity** |  |  |  |  |  |  |  |  |  |  |
| Whole-day – Post – CT | 1 | -1.65 [-2.2, -1.11] | * | * |  | * | * | * | * |  |
| Whole-day - Post - RCT | 7 | 0.27 [0.11, 0.42] | 0.00 | <.001 |  | 0.22 [-0.04, 0.43] | 0.87 | 1.44 | 0.14 [0.03, 0.36] |  |
| PA - Whole-day – Short-term - RCT | 1 | 0.19 [-0.37, 0.76] | * | * |  | * | * | * | * |  |
| PA - Whole-day – Long-term - RCT | 1 | -0.43 [-0.85, -0.01] | * | * |  | * | * | * | * |  |
| Sedentary behaviour |  |  |  |  |  |  |  |  |  |  |
| Whole-day - Post - RCT | 2 | 0.14 [-0.30, 0.57] | 42.98 | 0.545 |  | 0.02 [-0.73, 0.59] | 0.26 | 0.92 | 0.20 [0.04, 0.75] |  |
| Whole-day – Long-term – RCT | 1 | 0.00 [-0.37, 0.37] | * | * |  | * | * | * | * |  |
| **Community** |  |  |  |  |  |  |  |  |  |  |
| Whole-day – Post - CT | 1 | 0.81 [-0.11, 1.73] | * | * |  | * | * | * | * |  |
| **School & Family/Home** |  |  |  |  |  |  |  |  |  |  |
| **Physical activity** |  |  |  |  |  |  |  |  |  |  |
| PA - Post – Whole-day – CT | 1 | 0.05 [-0.44, 0.55] | * | * |  | * | * | * | * |  |
| PA - Post – Whole-day – RCT | 3 | 0.43 [0.21, 0.64] | 0.00 | <.001 |  | 0.32 [-0.27, 0.69] | 0.96 | 4.04 | 0.21 [0.04, 0.66] |  |
| PA - Short-term – Whole-day – CT | 1 | -0.23 [-0.72, 0.26] | * | * |  | * | * | * | * |  |
| PA - Short-term – Whole-day – RCT | 1 | 0.62 [0.35, 0.88] | * | * |  | * | * | * | * |  |
| **Sedentary behaviour** |  |  |  |  |  |  |  |  |  |  |
| SB – Whole-day – Post – RCT | 1 | -0.13 [-0.68, 0.28] | * | * |  | * | * | * | * |  |
| **Community & Family/Home** |  |  |  |  |  |  |  |  |  |  |
| **Physical activity** |  |  |  |  |  |  |  |  |  |  |
| PA – post – Whole day | 1 | 0.01 [-0.52, 0.55] | * | * |  | * | * | * | * |  |
| PA – Long-term - Whole day | 1 | 0.27 [-0.30, 0.84] | * | * |  | * | * | * | * |  |
| n=number of studies in meta-analysis; SMD=Standardized mean difference; CI95%=95% confidence interval; I2=Heterogeneity; ES=Effect size; CrI95%=Credible interval; PB BF10=Bayes factor (10) for publication bias; ES BF10=Bayes factor (10) for effect; τ BF10=Bayes factor (10) for heterogeneity; RCT=Randomized controlled trial; CT=(non-randomized) Controlled trial | | | | | | | | | | |
| P-values in bold digits indicate statistically significant effects. Bayes factors in bold digits indicate moderate evidence or higher. | | | | | | | | | | |


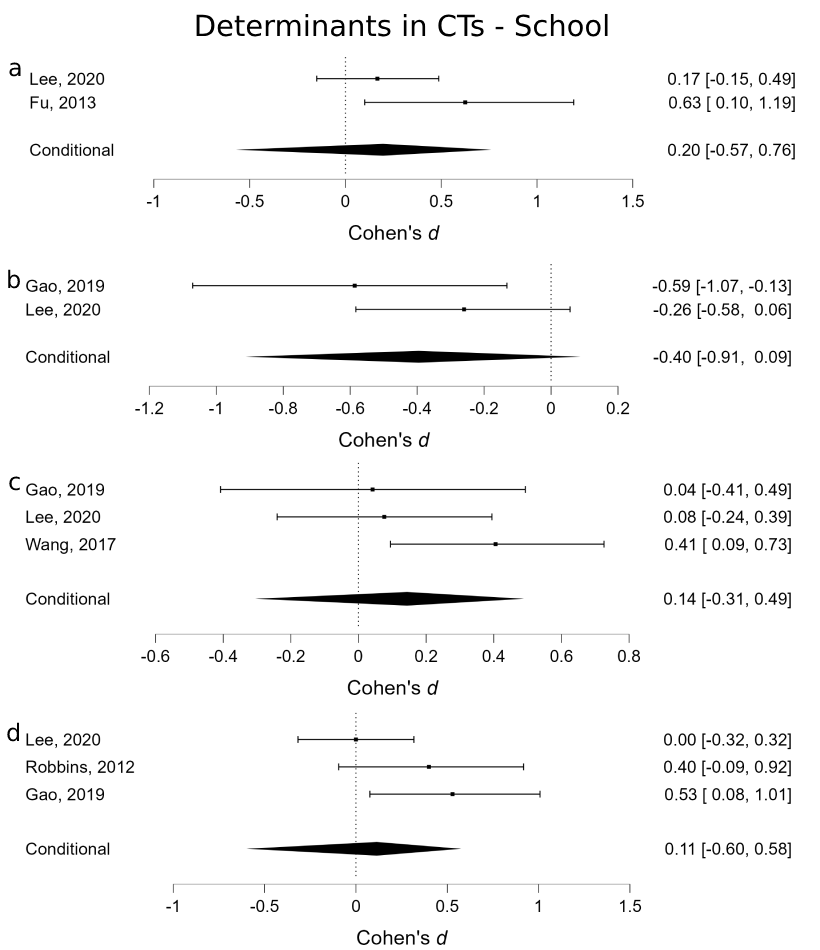


Figure S1. Forest plots for the post-intervention effect on determinants of PA/SB in CTs in the School setting: (a) Enjoyment; (b) PA outcome expectancies; (c) Self-efficacy; (d) Social support – General. School post CT. Includes post-intervention and short-term (<6 months post-intervention) effects.


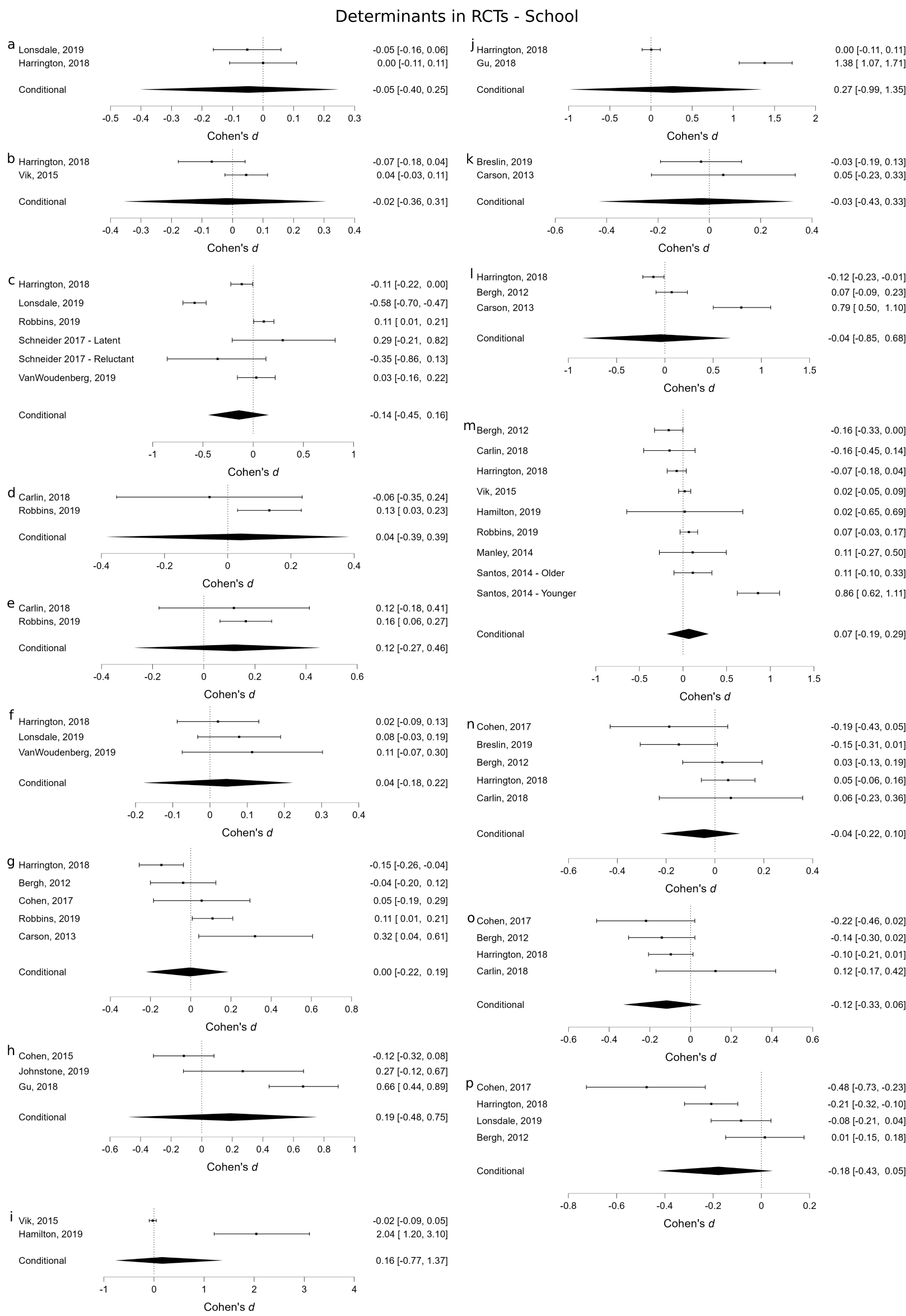


Figure S2. Forest plots for the post-intervention effect on determinants of PA/SB in RCTs in the School setting: (a) Amotivation, (b) Attitudes, (c) Autonomous motivation, (d) Barriers to PA, (e) Benefits of PA, (f) Controlled motivation, (g) Enjoyment, (h) Motor competence, (i) PA knowledge, (j) PA outcome expectancies, (k) Parenting for PA, (l) Perception of physical environment, (m) Self-efficacy, (n) Social support - Friends, (o) Social support - Parents, (p) Social support – Teachers.


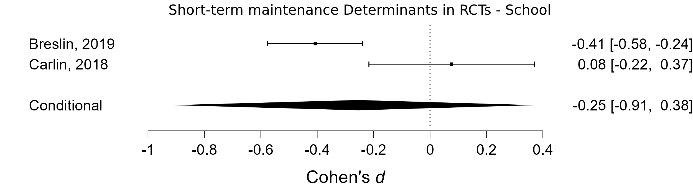


Figure S3. Forest plot for the short-term maintenance (<6 months post-intervention) on determinants of PA/SB in the School setting: Social support – Friends.


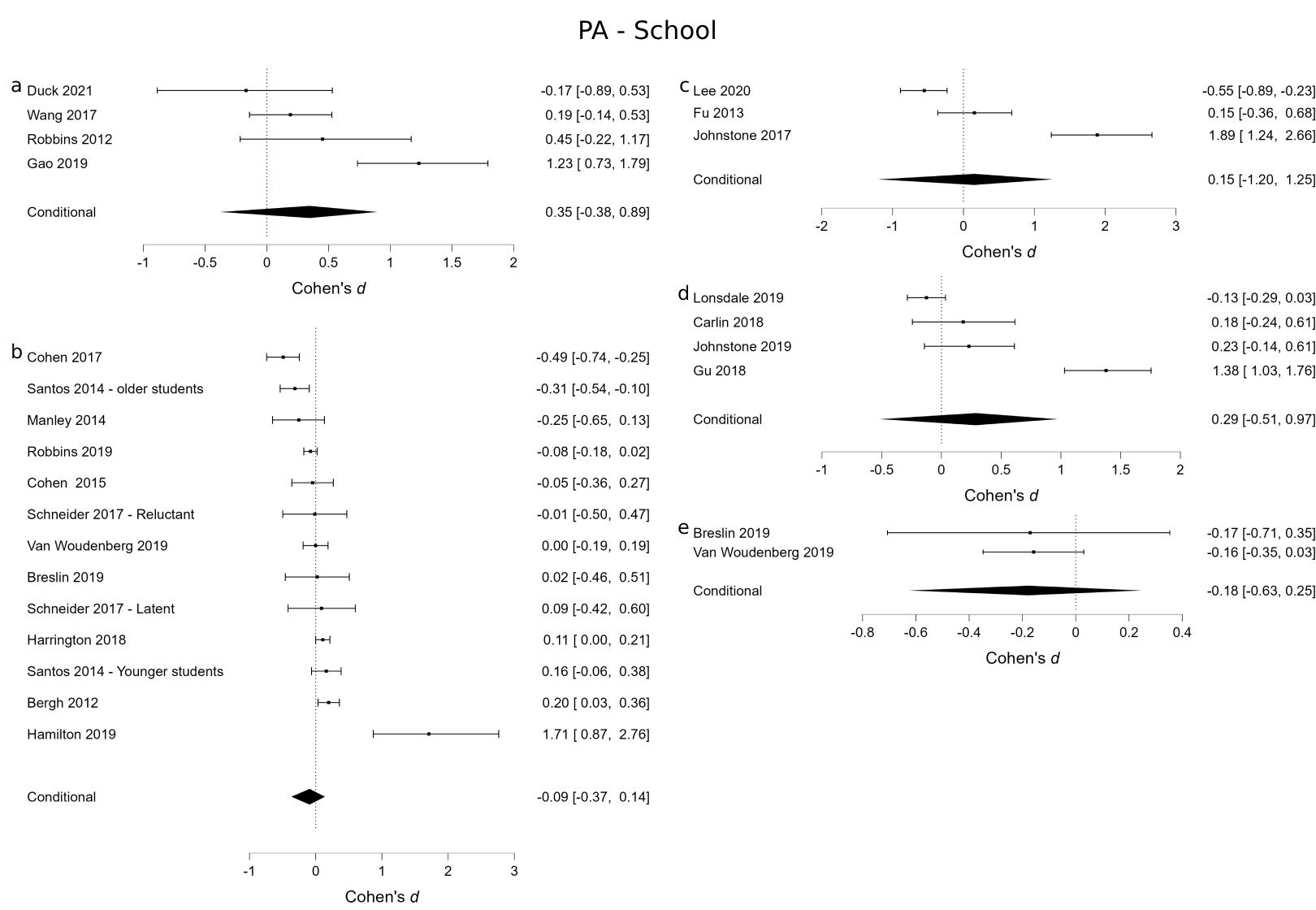


Figure S4. Forest plots for the effect on PA in the school setting in both RCTs and CTs: (a) Whole-day - Post – CT, (b) Whole-day - Post – RCT, (c) Part-day - Post – CT, (d) Part-day - Post – RCT, (e) Whole-day - Short-term – RCT. Includes post-intervention effects, and maintenance effects in the short-term (<6 months post-intervention) and long-term (>6 months post-intervention).


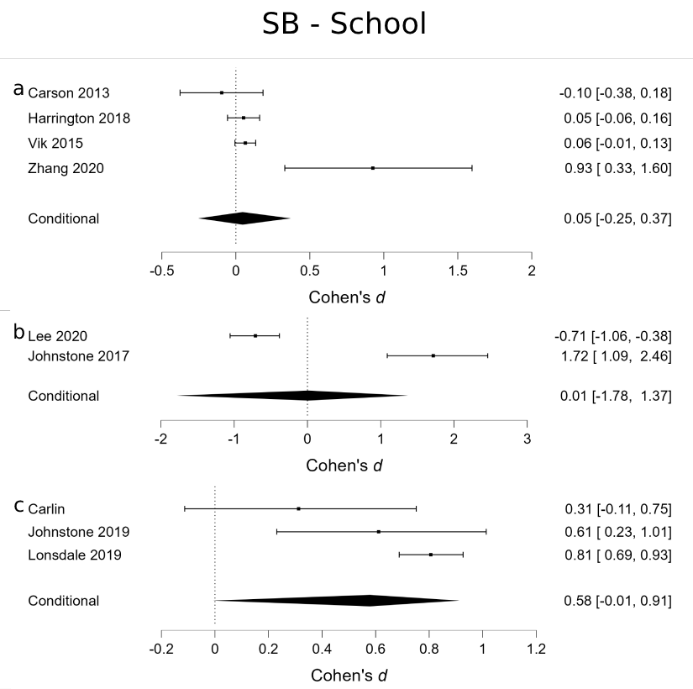


Figure S5. Forest plots for the effect on SB in the school setting in both RCTs and CTs: (a) Whole-day - Post – RCT, (b) Part-day - Post – CT, (c) Part-day - Post – RCT. Includes post-intervention effects.


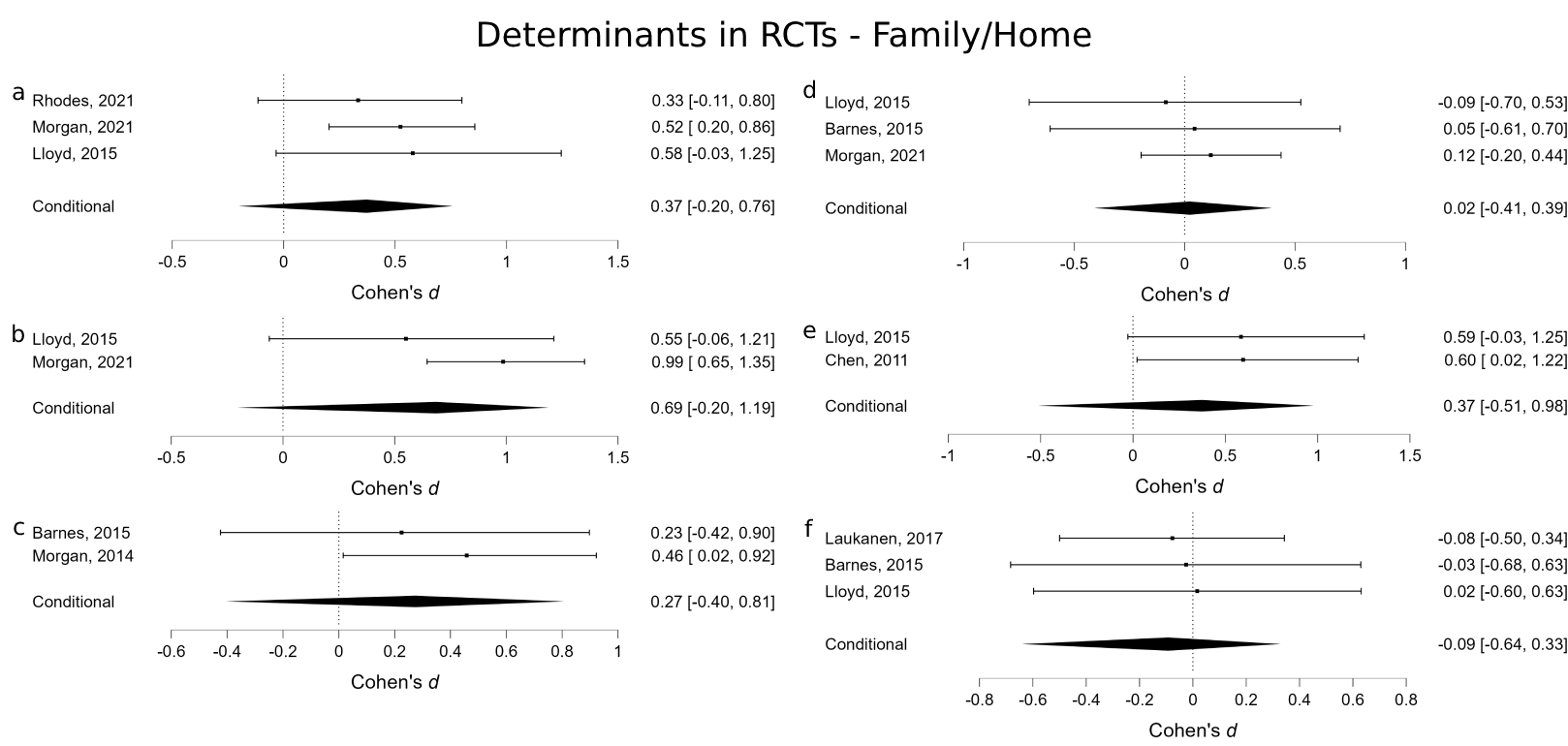


Figure S6. Forest plots for the post-intervention effect on determinants of PA/SB in RCTs in the Family/Home setting: (a) Co-PA, (b) Parental PA modelling, (c) Parental PA behaviour, (d) Parenting for PA, (e) Self-efficacy, (f) Social support – Parents. Includes post-intervention effects.


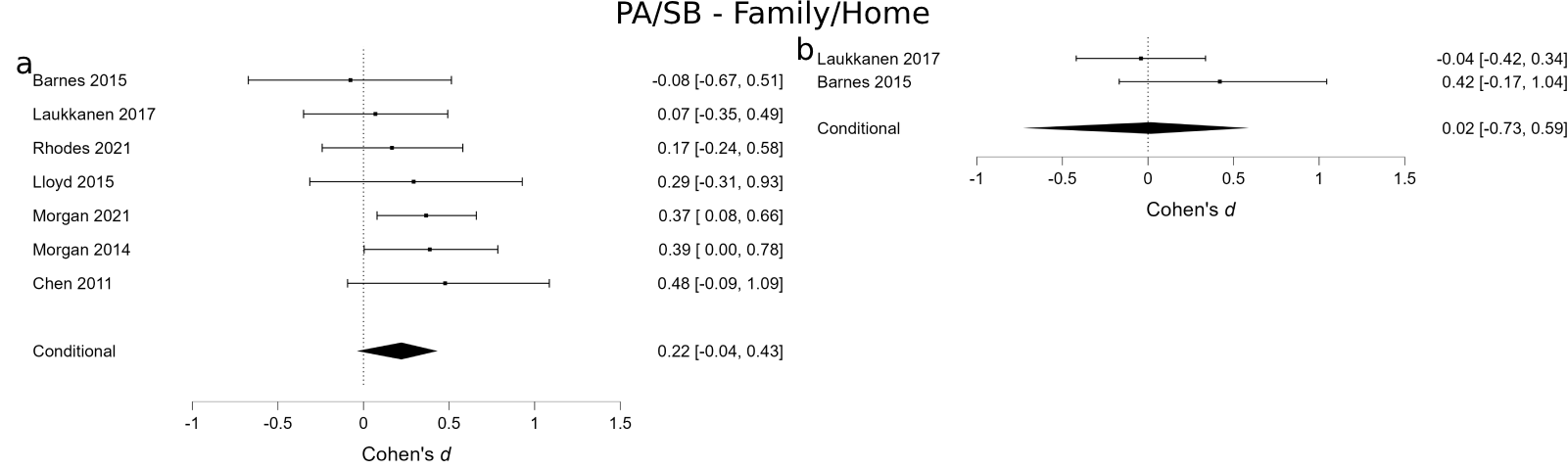


Figure S7. Forest plots for the effect on PA and SB in the Family/Home setting in RCTs: (a) PA Whole-day - Post – RCT, SB Whole-day - Post – RCT. Includes post-intervention effects.

**
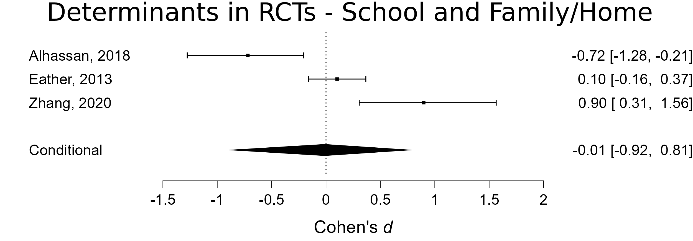
**

Figure S8. Forest plots for the post-intervention effect on determinants of PA/SB in RCTs in the combined School and Family/Home settings: Self-efficacy.


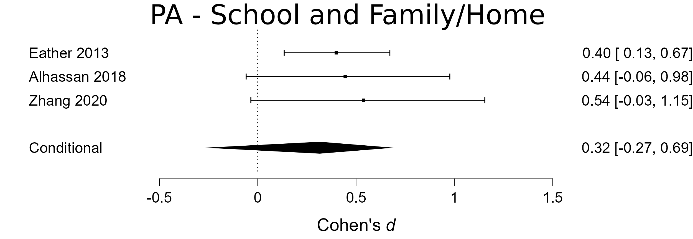


Figure S9. Forest plots for the effect on PA in the combined School and Family/Home setting in RCTs: Whole-day – Post – RCT.
