# Supplementary material for: DE-PASS Best Evidence Statement (BESt): A Systematic Review and Meta-analysis on the Effectiveness of Trials on Device-Measured Physical Activity and Sedentary Behaviour and Their Determinants in Children Aged 5–12 Years
Source: Sports Med. 2024 Dec 6;55(2):419–58. doi: 10.1007/s40279-024-02136-8 (PMC11947004; doi:10.1007/s40279-024-02136-8)
Supplement: Supplementary file 2 — GRADE assessments [file 40279_2024_2136_MOESM2_ESM.pdf]

Table S1. Determinants in the school setting.

Author(s):
Question: Intervention compared to Control for changing determinants in children 5-12 years in the school setting
Setting: School
Bibliography:

Certainty assessment							№ of patients		Effect		Certainty	Importance
**№ of studies**	**Study design**	**Risk of bias**	**Inconsistency**	**Indirectness**	**Imprecision**	**Other considerations**	**Intervention**	**Control**	**Relative (95% CI)**	**Absolute (95% CI)**		
**Enjoyment - CT - Post**												
2	non-randomised studies	very serious[a]	not serious[b]	not serious	serious[c]	all plausible residual confounding would reduce the demonstrated effect	108	110	-	mean **0.2 SD higher** (0.57 lower to 0.76 higher)	⊕⊕◯◯ Low	
**PA outcome expectancies - CT - Post**												
2	non-randomised studies	very serious[d]	not serious[e]	not serious	serious[f]	all plausible residual confounding would reduce the demonstrated effect	122	116	-	mean **0.4 lower** (0.91 lower to 0.09 higher)	⊕⊕◯◯ Low	
**Self-efficacy - CT - Post**												
3	non-randomised studies	very serious[g]	not serious[h]	not serious	serious[i]	all plausible residual confounding would reduce the demonstrated effect	212	192	-	mean **0.14 higher** (0.31 lower to 0.49 higher)	⊕⊕◯◯ Low	
**Social support - General - CT - Post**												
3	non-randomised studies	very serious[j]	not serious[k]	not serious	serious[l]	all plausible residual confounding would reduce the demonstrated effect	158	147	-	mean **0.11 higher** (0.6 lower to 0.58 higher)	⊕⊕◯◯ Low	
**Amotivation - RCT - Post**												
2	randomised trials	not serious[m]	not serious[n]	not serious	very serious[o]	none	1360	1198	-	mean **0.05 lower** (0.4 lower to 0.25 higher)	⊕⊕◯◯ Low	
**Attitudes - RCT - Post**												
2	randomised trials	not serious[p]	not serious[q]	not serious	very serious[r]	none	2306	2151	-	mean **0.02 lower** (0.36 lower to 0.31 higher)	⊕⊕◯◯ Low	
**Autonomous motivation - RCT - Post**												
6	randomised trials	not serious[s]	serious[t]	not serious	serious[u]	none	2432	2222	-	mean **0.14 lower** (0.45 lower to 0.16 higher)	⊕⊕◯◯ Low	
**Barriers to PA - RCT - Post**												
2	randomised trials	not serious[v]	not serious[w]	not serious	very serious[x]	none	849	853	-	mean **0.04 higher** (0.39 lower to 0.39 higher)	⊕⊕◯◯ Low	
**Benefits of PA - RCT - Post**												
2	randomised trials	not serious[y]	not serious[z]	not serious	very serious[aa]	none	849	853	-	mean **0.12 higher** (0.27 lower to 0.46 higher)	⊕⊕◯◯ Low	
**Controlled motivation - RCT - Post**												
3	randomised trials	not serious[ab]	not serious[ac]	not serious	very serious	none	1610	1384	-	mean **0.04 higher** (0.18 lower to 0.22 higher)	⊕⊕◯◯ Low	
**Enjoyment - RCT - Post**												
5	randomised trials	serious[ad]	not serious[ae]	not serious	serious[af]	none	2100	1989	-	mean **0** (0.22 lower to 0.19 higher)	⊕⊕◯◯ Low	
**Motor competence - RCT - Post**												

3	randomised trials	very serious[ag]	serious[ah]	not serious	serious[ai]	none	737	746	-	mean **0.19 higher** (0.48 lower to 0.75 higher)	⨁◯◯◯ Very low	
**PA knowledge - RCT - Post**												
2	randomised trials	serious[aj]	not serious[ak]	not serious	very serious[al]	none	1589	1597	-	mean **0.16 higher** (0.77 lower to 1.37 higher)	⨁◯◯◯ Very low	
**PA outcome expectancies - RCT - Post**												
2	randomised trials	not serious[am]	serious[an]	not serious	serious[ao]	none	929	630	-	mean **0.27 higher** (1 lower to 1.35 higher)	⨁⨁◯◯ Low	
**Parenting for PA - RCT - Post**												
2	randomised trials	not serious[ap]	not serious[aq]	not serious	very serious[ar]	none	527	386	-	mean **0.03 lower** (0.43 lower to 0.33 higher)	⨁⨁◯◯ Low	
**Perception of physical environment - RCT - Post**												
3	randomised trials	not serious[as]	serious[at]	not serious	serious[au]	none	1173	1118	-	mean **0.04 lower** (0.86 lower to 0.68 higher)	⨁⨁◯◯ Low	
**Self-efficacy - RCT - Post**												
9	randomised trials	not serious[av]	not serious[aw]	not serious	very serious[ax]	none	3771	3868	-	mean **0.07 higher** (0.19 lower to 0.29 higher)	⨁⨁◯◯ Low	
**Social support - Friends - RCT - Post**												
5	randomised trials	not serious[ay]	not serious[az]	not serious	very serious[ba]	none	1522	1564	-	mean **0.04 lower** (0.22 lower to 0.1 higher)	⨁⨁◯◯ Low	
**Social support - Parents - RCT - Post**												
4	randomised trials	serious[bb]	not serious[bc]	not serious	very serious[bd]	none	1218	1251	-	mean **0.12 lower** (0.33 lower to 0.06 higher)	⨁◯◯◯ Very low	
**Social support - Teachers - RCT - Post**												
4	randomised trials	serious[be]	serious[bf]	not serious	serious[bg]	none	1734	1791	-	mean **0.18 lower** (0.43 lower to 0.05 higher)	⨁◯◯◯ Very low	
**Social support - Friends - RCT - Short-term**												
2	randomised trials	not serious[bh]	serious[bi]	not serious	serious[bj]	none	397	357	-	mean **0.25 lower** (0.91 lower to 0.38 higher)	⨁⨁◯◯ Low	

CI: confidence interval

Explanations

a. Both studies judged as serious risk. Serious risk for both studies in domains 1 (confounding variables), 6 (measurement of the outcome) and 7 (measurement of the determinants).
b. Overlapping CIs, small variation in estimates, anecdotal evidence for the presence of heterogeneity as indicated by BF10.
c. CI is wide; anecdotal evidence for the presence of an effect as indicated by BF10.
d. Both studies judged as serious risk. Serious risk for both studies in domains 1 (confounding variables), 6 (measurement of the outcome) and 7 (measurement of the determinants).
e. Overlapping CIs, small variation in estimates, moderate evidence for the lack of heterogeneity as indicated by BF10.
f. CI is wide; anecdotal evidence for the presence of an effect as indicated by BF10.
g. Both studies judged as serious risk. Serious risk for both studies in domains 1 (confounding variables), 6a (measurement of the outcome) and 6b (measurement of the determinants).
h. Overlapping CIs, small variation in estimates, anecdotal evidence for the presence of heterogeneity as indicated by BF10.
i. CI is wide; anecdotal evidence for the presence of an effect as indicated by BF10.
j. All three studies judged as serious risk. Serious risk for all three studies in domain 1 (confounding variables). For Robbins (2012), moderate risk in domains 3 (classification of interventions), 6 (measurement of the outcome) and 7 (measurement of the determinants). For Gao (2019) and Lee (2020), serious risk on domains 6 and 7.
k. Overlapping CIs, small variation in estimates, anecdotal evidence for the presence of heterogeneity as indicated by BF10.
l. CI is wide; anecdotal evidence for the presence of an effect as indicated by BF10.
m. Both studies judged as low risk.
n. Overlapping CIs, small variation in estimates, moderate evidence for the lack of heterogeneity as indicated by BF10.
o. CI is wide; moderate evidence for the lack of evidence as indicated by BF10.
p. Harrington (2018) was judged as low risk. Vik (2015) was judged as some concerns in domains 1 (randomization process) and domain 5(measurement of the determinants).
q. Overlapping CIs, small variation in estimates, anecdotal evidence for the presence of heterogeneity as indicated by BF10.
r. CI is wide; anecdotal evidence for the presence of an effect as indicated by BF10.
s. Two of five studies judged as high risk. Robbins (2019) high risk due to some concerns in domains 1 (randomization process), 5 (measurement of the outcome) and 6 (selection of the reported result). Van Woudenberg (2019) high risk due to some concerns in domains 1, 2 (deviations from the intended interventions) and 3 (missing outcome data).
t. Mostly overlapping CIs, moderate variation in estimates, extreme evidence for the presence of heterogeneity as indicated by BF10.

u. CI is wide; anecdotal evidence for the presence of an effect as indicated by BF10.
v. Carlin judged as some concerns while Robbins judged as high risk. Both studies judged as some concerns in domains 5 (measuremernt of the outcome) and 6 (selection of the reported result). Robbins (2019) also judged as some concerns on domain 1 (randomization process).
w. Overlapping CIs, moderate variation in estimates, anecdotal evidence for the presence of heterogeneity as indicated by BF10.
x. CI is wide; anecdotal evidence for the presence of an effect as indicated by BF10.
y. Carlin judged as some concerns while Robbins judged as high risk. Both studies judged as some concerns in domains 5 (measuremernt of the outcome) and 6 (selection of the reported result). Robbins (2019) also judged as some concerns on domain 1 (randomization process).
z. Overlapping CIs, small variation in estimates, anecdotal evidence for the presence of heterogeneity as indicated by BF10.
aa. CI is wide; anecdotal evidence for the presence of an effect as indicated by BF10.
ab. Harrington (2018) and Lonsdale (2019) judged as low risk. Van Woudenberg (2019) judged as high risk due to some concerns in domains 1 (randomization process), 2 (deviations from the intended interventions) and 3 (missing outcome data).
ac. Overlapping CIs, small variation in estimates, moderate evidence for the lack of heterogeneity as indicated by BF10.
ad. Harrington (2018) was judged as low risk, Carson (2013) was judged as some concerns in domains 2 (deviations from the intended interventions) and 4 (measurement of the outcome). Bergh (2012) and Cohen (2017) judged as high risk due to high risk in domains 2 and 5, respectively. Robbins (2019) judged as some concerns in domains 1 (randomization process), 4, and 6 (selection of the reported result).
ae. Mostly overlapping CIs, small variation in estimates, anecdotal evidence for the presence of heterogeneity as indicated by BF10.
af. CI is wide; anecdotal evidence for the presence of an effect as indicated by BF10.
ag. All three studies judged as high risk. Gu (2018) and Johnstone (2019) judged as high risk in domain 2 (deviations from the intended interventions). Cohen (2015) judged as some concern in domains 2, 3 (missing outcome data), and 6 (selection of the reported result).
ah. Mostly overlapping CIs, wide variation in estimates, extreme evidence for the presence of heterogeneity as indicated by BF10.
ai. CI is wide; anecdotal evidence for the presence of an effect as indicated by BF10.
aj. Vik was judged as some concerns with some concerns judgements in domains 1 (randomization process) and 4 (measurement of the outcome). Hamilton was judged as high risk due to some concerns on domains 1, 3 (missing data) and 4. Additionally, Hamilton was judged as high risk in domain 6 (selection of the reported results).
ak. No overlap in CIs, wide variation in estimates, anecdotal evidence for the presence of heterogeneity as indicated by BF10. Vik (2015) has very large sample size compared to Hamilton (2019), thus higher weight.
al. CI is wide; anecdotal evidence for the presence of an effect as indicated by BF10.
am. Harrington (2018) judged as low risk. Gu (2018) judged as high risk due to high risk in domain 2 (deviations from the intended interventions) and some concerns in domains 4 (measurement of the outcome), 5 (measurement of the determinants) and 6 (selection of the reported result).
an. No overlap in CIs, wide variation in estimates, strong evidence for the presence of heterogeneity as indicated by BF10.
ao. CI is wide; anecdotal evidence for the presence of an effect as indicated by BF10.
ap. Both studies judged as some concerns. Breslin (2019) due to some concerns in domains 1 (randomization process) and 3 (missing outcome data). Carson (2013) due to high rik in domain 2 (deviations from the intended interventions) and 5 (measurement of the determinants).
aq. Overlapping CIs, small variation in estimates, moderate evidence for the lack of heterogeneity as indicated by BF10.
ar. CI is wide; moderate evidence for the lack of evidence as indicated by BF10.
as. Harrington (2018) judged as low risk. Bergh (2012) some concerns in domain 1 (randomization process) and high risk in domain 2 (deviations from the intended interventions). Carson (2013) some concerns in domain 2 and some concerns in domain 5 (measurement of the determinants).
at. Mostly overlapping CIs, moderate variation in estimates, moderate evidence for the presence of heterogeneity as indicated by BF10.
au. CI is wide; anecdotal evidence for the presence of an effect as indicated by BF10.
av. Bergh (2012), Hamilton (2019) and Manley (2014) high risk in domains 2 (deviation from intended intervention), 6 (selection of the reported results), and 1 (randomization process) and 3 (missing data), respectiveley. Robbins (2019) high risk due to some concerns in domains 1, 5 (measurement of the determinant) and 6. Carlin (2018) and Santos (2014) some concerns due to some concerns in domains 5 and 6; and Vik (2015) some concerns due to some concerns in domains 1 and 5. Harrington (2018) low risk.
aw. Mostly overlapping CIs, small variation in estimates, extreme evidence for the presence of heterogeneity as indicated by BF10. - Santos (2014) - Younger group sticks out.
ax. CI is wide; moderate evidence for the lack of evidence as indicated by BF10.
ay. Harrington (2018) low risk. Breslin (2019) and Carlin (2018) both some concerns in domains 1 (randomization process) and 3 (missing data), and 5 (measurement of the determinant) and 6 (measurement of the outcomes), respectively. Bergh (2012) and Cohen (2017) high risk in domains 2 (deviation from intended intervention) and 5, respectively.
az. Overlapping CIs, small variation in estimates, anecdotal evidence for the presence of heterogeneity as indicated by BF10.
ba. CI is wide; moderate evidence for the lack of evidence as indicated by BF10.
bb. Harrington (2018) low risk. Carlin (2018) some concerns in domains 5 (measurement of the determinants) and 6 (selection of the reported result). Berg (2012) and Cohen (2017) high risk in domains 2 (deviation from intended intervention) and 5.
bc. Overlapping CIs, small variation in estimates, anecdotal evidence for the presence of heterogeneity as indicated by BF10.
bd. CI is wide; moderate evidence for the lack of evidence as indicated by BF10.
be. Harrington (2018) and Lonsdale (2019) judged as low risk. Berg (2012) some concerns in domain 1 (randomization process) and high risk in domain 2 deviations from the intended interventions). Cohen (2017) some concerns in domain 1 and high risk in domain 5 (measurement of the determinants).
bf. Mostly overlapping CIs, moderate variation in estimates, strong evidence for the presence of heterogeneity as indicated by BF10.
bg. CI is wide; anecdotal evidence for the presence of an effect as indicated by BF10.
bh. Both studies judged as some concerns. Breslin (2019) some concerns in domains 1 (randomization process) and 3 (missing outcome data). Carlin (2018) some concerns in domains 5 (measurment of the determinants) and 6 (selection of the reported result).
bi. No overlap in CIs, moderate variation in estimates, moderate evidence for the presence of heterogeneity as indicated by BF10.
bj. CI is wide; anecdotal evidence for the presence of an effect as indicated by BF10.

Table S2. Physical activity in the school setting.

Author(s):
Question: Intervention compared to Control for increasing physical activity in children 5-12 years in the school setting
Setting: School
Bibliography:

Certainty assessment							№ of patients		Effect		Certainty	Importance
№ of studies	Study design	Risk of bias	Inconsistency	Indirectness	Imprecision	Other considerations	Intervention	Control	Relative (95% CI)	Absolute (95% CI)		
**Physical activity - CT - Post (assessed with: Whole-day)**												
4	non-randomised studies	very serious[a]	serious[b]	not serious	serious[c]	all plausible residual confounding would reduce the demonstrated effect	156	143	-	mean **0.35 SD higher** (0.38 lower to 0.89 higher)	⊕◯◯◯ Very low	
**Physical activity - RCT - Post (assessed with: Whole-day)**												
13	randomised trials	serious[d]	serious[e]	not serious	not serious[f]	none	2735	2735	-	mean **0.09 lower** (0.37 lower to 0.14 higher)	⊕⊕◯◯ Low	
**Physical activity - CT - Post (assessed with: Part-day)**												
3	non-randomised studies	very serious[g]	very serious[h]	not serious	serious[i]	all plausible residual confounding would reduce the demonstrated effect	171	128	-	mean **0.15 SD higher** (1.2 lower to 1.25 higher)	⊕◯◯◯ Very low	
**Physical activity - RCT - Post (assessed with: Part-day)**												
4	randomised trials	serious[j]	serious[k]	not serious	serious[l]	none	656	395	-	mean **0.29 higher** (0.51 lower to 0.97 higher)	⊕◯◯◯ Very low	
**Physical activity - RCT - Short-term (assessed with: Whole-day)**												
2	randomised trials	serious[m]	not serious[n]	not serious	not serious[o]	none	287	219	-	mean **0.18 lower** (0.63 lower to 0.25 higher)	⊕⊕⊕◯ Moderate	

CI: confidence interval

Explanations

a. All studies in the meta-analysis judged as serious risk, mainly in domains 1 (confounding variables), 6(measurement of outcome) and 7(measurement of determinants). Robbins (2019) judged as moderate risk in domains 6 and 7.
b. Mostly overlapping CIs, moderate variation in estimates, strong evidence for the presence of heterogeneity as indicated by BF10.
c. CI is wide; anecdotal evidence for the presence of an effect as indicated by BF10.
d. All studies were judged as high risk of bias, except Harrington (2018) and Schneider (2017), judged as low risk of bias and Santos (2014) and Breslin (2019), judged as some concerns. High risk was due to some concerns and high risk on two or more domains (no specific domain sticks out). Domain 4 (measurement of outcome; PA/SB) was judged as low risk for all studies.
e. Mostly overlapping CIs, low variation in estimates except for Hamilton (2019), extreme evidence for the presence of heterogeneity as indicated by BF10.
f. CI is narrow; anecdotal evidence for the presence of an effect as indicated by BF10.
g. All three studies judged as serious risk. Johnstone (2017) judged as serious risk on all domains except domains 2 (deviations from intended intervention) and 7 (selection of the reported result), which were judged as no information (NI).
h. Partially overlapping CIs, wide variation in estimates, extreme evidence for the presence of heterogeneity as indicated by BF10.
i. CI is wide; anecdotal evidence for the presence of an effect as indicated by BF10.
j. Lonsdale (2019) judged as low risk. Carlin (2018) some concerns in domains 4 and 5 (measurement of the outcome, and determinants, respectively). For Gu (2018) and Johnstone (2019), high risk was due to high risk in domain 2 (deviations from the intended interventions).
k. Mostly overlapping CIs, moderate variation in estimates, extreme evidence for the presence of heterogeneity as indicated by BF10.
l. CI is wide; anecdotal evidence for the presence of an effect as indicated by BF10.
m. Breslin (2019) some concerns in domains 1 (the randomization process) and 3 (missing outcome data). Van Woudenberg (2019) jusdged as high risk dues to some concerns in domain 2 (deviations from the intended interventions).
n. Overlapping CIs, consistent estimates, anecdotal evidence for the presence of heterogeneity as indicated by BF10.
o. CI is moderately wide; anecdotal evidence for the presence of an effect as indicated by BF10.

Table S3. Sedentary behaviour in the school setting.

Author(s):
Question: Intervention compared to Control for reducing sedentary behaviour in children 5-12 years in the school setting
Setting: School
Bibliography:

Certainty assessment							№ of patients		Effect		Certainty	Importance
№ of studies	Study design	Risk of bias	Inconsistency	Indirectness	Imprecision	Other considerations	Intervention	Control	Relative (95% CI)	Absolute (95% CI)		
**Sedentary behaviour - RCT - Post (assessed with: Whole-day)**												
4	randomised trials	not serious[a]	not serious[b]	not serious	serious[c]	none	2563	2248	-	mean **0.05 higher** (0.25 lower to 0.37 higher)	⨁⨁⨁◯ Moderate	
**Sedentary behaviour - CT - Post (assessed with: Part-day)**												
2	non-randomised studies	very serious[d]	very serious[e]	not serious	very serious[f]	all plausible residual confounding would reduce the demonstrated effect	140	98	-	mean **0.01 higher** (1.78 lower to 1.37 higher)	⨁◯◯◯ Very low	
**Sedentary behaviour - RCT - Post (assessed with: Part-day)**												
3	randomised trials	not serious[g]	not serious[h]	not serious	not serious[i]	none	748	718	-	mean **0.58 higher** (0.01 lower to 0.91 higher)	⨁⨁⨁⨁ High	

CI: confidence interval

Explanations

a. Harrington (2018) judged as low risk. Carson (2013), Vik (2015) and Zhang (2020) were judged as some concerns on domain 2 (deviations from the intended interventions) and 5 (measurement of the outcome), 1 (randomization process) and 6 (selection of the reported results), and 2 and 6, respectively.
b. Mostly overlapping CIs except for Zhang (2020), moderate variation in estimates, anecdotal evidence for the presence of heterogeneity as indicated by BF10.
c. CI is narrow; moderate evidence for the lack of effect as indicated by BF10.
d. Both studies judged as serious risk. Serious risk on both studies was due to serious risk on domains 1 (confounding variables), 6 (measurement of outcomes) and 7(measurement of determinants). Additionally, Jonstone (2017) was serious risk on the remaining domains.
e. No overlap in CIs, wide variation in estimates, very strong evidence for the presence of heterogeneity as indicated by BF10.
f. CI is wide; anecdotal evidence for the presence of an effect as indicated by BF10.
g. Lonsdale (2019) judged as low risk. Carlin (2018) judged as some concerns on domains 5 and 6 (measurement of the determinant(s) and outcomes, respectively). Johnstone (2019) judged as high risk on somain 2 (deviation from the intended intervention).
h. Overlapping CIs, small variability in estimates, anecdotal evidence for the presence of heterogeneity as indicated by BF10.
i. CI is moderately wide; moderate evidence for the presence of an effect as indicated by BF10.

Table S4. Determinants in the family/home setting.

Author(s):
Question: Intervention compared to Control for changing determinants in children 5-12 years in the family/home setting
Setting: Family/Home
Bibliography:

Certainty assessment							№ of patients		Effect		Certainty	Importance
**№ of studies**	**Study design**	**Risk of bias**	**Inconsistency**	**Indirectness**	**Imprecision**	**Other considerations**	**Intervention**	**Control**	**Relative (95% CI)**	**Absolute (95% CI)**		
**Co-PA - RCT - Post**												
3	randomised trials	not serious[a]	not serious[b]	not serious	serious[c]	none	143	140	-	**0.37 higher** (0.2 lower to 0.76 higher)	⨁⨁⨁◯ Moderate	
**Parental modeling - RCT - Post**												
2	randomised trials	not serious[d]	not serious[e]	not serious	not serious[f]	none	101	102	-	mean **0.69 higher** (0.2 lower to 1.19 higher)	⨁⨁⨁⨁ High	
**Parental PA behaviour - RCT - Post**												
2	randomised trials	serious[g]	not serious[h]	not serious	serious[i]	none	63	60	-	**0.27 higher** (0.4 lower to 0.81 higher)	⨁⨁◯◯ Low	
**Parenting for PA - RCT - Post**												
3	randomised trials	not serious[j]	not serious[k]	not serious	serious[l]	none	122	121	-	**0.02 higher** (0.41 lower to 0.39 higher)	⨁⨁⨁◯ Moderate	
**Self-efficacy - RCT - Post**												
2	randomised trials	not serious[m]	not serious[n]	not serious	serious[o]	none	49	47	-	**0.37 higher** (0.51 lower to 0.98 higher)	⨁⨁⨁◯ Moderate	
**Social support - Parents - RCT - Post**												
3	randomised trials	serious[p]	not serious[q]	not serious	serious[r]	none	88	88	-	**0.09 lower** (0.64 lower to 0.33 higher)	⨁⨁◯◯ Low	

CI: confidence interval

Explanations

a. Lloyd (2015) and Morgan (2021) judged as low risk. Rhodes (2021) judged as some concerns in domain 5 (measurement of the determinants).
b. Overlapping CIs, small variation in estimates, anecdotal evidence for the presence of heterogeneity as indicated by BF10.
c. Wde CI and anecdotal evidence for the effectg of the intervention on the determinant indicated by BF10.
d. Risk of bias was judged as low risk for both studies in the meta-analysis.
e. Overlapping CIs, small variation in estimates, anecdotal evidence for the presence of heterogeneity as indicated by BF10.
f. CI is wide; moderate evidence for the presence of an effect as indicated by BF10.
g. Barnes (2015) judged as some concerns in domain 6 (selection of the reported result). Morgan (2014) judged as high risk due to some concerns in domains 4 (measurement of the outcome) and 5 (measurement of the determinants).
h. Overlapping CIs, small variation in estimates, anecdotal evidence for the presence of heterogeneity as indicated by BF10.
i. Wde CI and anecdotal evidence for the effect of the intervention on the determinant indicated by BF10.
j. Lloyd (2015) and Morgan (2021) judged as low risk. Barnes (2015) judged as some concerns in domain 6 (selection of the reported result).
k. Overlapping CIs, small variation in estimates, anecdotal evidence for the presence of heterogeneity as indicated by BF10.
l. Wde CI and anecdotal evidence for the effectg of the intervention on the determinant indicated by BF10.
m. Lloyd (2015) judged as low risk. Chen (2011) judged as some concerns in domain 1 (randomization process).
n. Overlapping CIs, small variation in estimates, anecdotal evidence for the presence of heterogeneity as indicated by BF10.
o. Wde CI and anecdotal evidence for the effectg of the intervention on the determinant indicated by BF10.
p. Lloyd (2015) judged as low risk. Barnes (2015) judged as some concerns in domain 6 (selection of the reported result). Laukkanen (2017) judged as high risk due to some concerns in domains 1 (randomization process) and 6.
q. Overlapping CIs, small variation in estimates, anecdotal evidence for the presence of heterogeneity as indicated by BF10.
r. Wde CI and anecdotal evidence for the effectg of the intervention on the determinant indicated by BF10.

Table S5. Physical activity in the family/home setting.

Author(s):
Question: Intervention compared to Control for increasing physical activity in children 5-12 years in the family/home setting
Setting: Family/Home
Bibliography:

Certainty assessment							№ of patients		Effect		Certainty	Importance
№ of studies	Study design	Risk of bias	Inconsistency	Indirectness	Imprecision	Other considerations	Intervention	Control	Relative (95% CI)	Absolute (95% CI)		
**Physical activity - RCT - Post (assessed with: Whole-day)**												
7	randomised trials	serious[a]	not serious[b]	not serious	not serious[c]	none	318	315	-	**0.22 higher** (0.04 lower to 0.43 higher)	⨁⨁⨁◯ Moderate	

CI: confidence interval

Explanations

a. Lloyd (2015) and Morgan (2021) judged as low risk. Barnes (2015), Chen (2011) and Rhodes (2021) judged as some concerns in domains 6 (slection of the reported result), 1 (randomization process), 5 (measurement of the determinants), respectively. Laukkanen (2017) judged as high risk due to some concerns in domains 1 and 6. Morgan (2014) judged as high risk due to some concerns in domains 4 (measurement of the outcome) and 5.
b. Overlapping CIs, small variation in estimates, anecdotal evidence for the presence of heterogeneity as indicated by BF10.
c. CI is narrow; anecdotal evidence for the presence of an effect as indicated by BF10.

Table S6. Sedentary behaviour in the family/home setting.

Author(s):
Question: Intervention compared to Control for reducing sedentary behaviour in children5-12 years in the family/home setting
Setting: Family/Home
Bibliography:

Certainty assessment							№ of patients		Effect		Certainty	Importance
**№ of studies**	**Study design**	**Risk of bias**	**Inconsistency**	**Indirectness**	**Imprecision**	**Other considerations**	**Intervention**	**Control**	**Relative (95% CI)**	**Absolute (95% CI)**		
**Sedentary behaviour - RCT - Post (assessed with: Whole-day)**												
2	randomised trials	serious[a]	not serious[b]	not serious	serious[c]	none	69	100	-	mean **0.02 higher** (0.73 lower to 0.59 higher)	⊕⊕◯◯ Low	

CI: confidence interval

Explanations

a. Barnes (2015) judged as some concerns in domain 6 (selection of the reported result). Laukkanen (2017) judged as high risk due to some concerns in domains 1 (randomization process) and 6.
b. Overlapping CIs, small variation in estimates, moderate evidence for the lack of heterogeneity as indicated by BF10.
c. CI is wide; anecdotal evidence for the presence of an effect as indicated by BF10.

Table S7. Determinants in the combined school and family/home setting.

Author(s):
Question: Intervention compared to Control for changing determinants in children 5-12 years in the combined school and family/home settings
Setting: School and Family/Home
Bibliography:

Certainty assessment							№ of patients		Effect		Certainty	Importance
№ of studies	Study design	Risk of bias	Inconsistency	Indirectness	Imprecision	Other considerations	Intervention	Control	Relative (95% CI)	Absolute (95% CI)		
**Self-efficacy - RCT - Post**												
3	randomised trials	not serious[a]	very serious[b]	not serious	very serious[c]	none	195	158	-	**0.01 lower** (0.92 lower to 0.81 higher)	⨁◯◯◯ Very low	

CI: confidence interval

Explanations

a. Alhassan (2018) judged as some concerns in domain 6 (selection of the reported result). Eather (2013) judged as some conerns in domains 4 (measurement of the outcome) and 5 (measurement of the determinants). Zhang (2020) judged as some concerns in domains 2 (deviations from the intended interventions) and domain 5.
b. Minimal overlap between CIs and a wide variation in estmates between studies in the meta-analysis. BF10 indicates moderate support for the lack of evidence for the effect.
c. Wide confidence interval around the estimate. Limits the confidence in the effect to be used as recommendation.

Table S8. Physical activity in the combined school and family/home setting.

Author(s):
Question: Intervention compared to Control for increasing physical activity in children 5-12 years in the combined school and family/home settings
Setting: School and Family/Home
Bibliography:

Certainty assessment							№ of patients		Effect		Certainty	Importance
№ of studies	Study design	Risk of bias	Inconsistency	Indirectness	Imprecision	Other considerations	Intervention	Control	Relative (95% CI)	Absolute (95% CI)		
**Physical activity - RCT - Post (assessed with: Whole-day)**												
3	randomised trials	not serious[a]	not serious[b]	not serious	not serious[c]	none	195	158	-	mean **0.32 higher** (0.27 lower to 0.69 higher)	⨁⨁⨁⨁ High	

CI: confidence interval

Explanations

a. Alhassan (2018) judged as some concerns in domain 5 (selection of the reported result). Eather (2013) some conerns in domains 4 (measurement of the outcome) and 5 (measurement of the determinants). Zhang (2020) judged as some concerns in domains 2 (deviations from the intended interventions) and 5.
b. Overlapping CIs, small variation in estimates, anecdotal evidence for the presence of heterogeneity as indicated by BF10.
c. CI is moderate; anecdotal evidence for the presence of an effect as indicated by BF10.
